# Carboxylic acids and light interact to affect nanoceria stability and dissolution in acidic aqueous environments

**DOI:** 10.3762/bjnano.14.63

**Published:** 2023-06-27

**Authors:** Matthew L Hancock, Eric A Grulke, Robert A Yokel

**Affiliations:** 1 Chemical and Materials Engineering, University of Kentucky, Lexington, KY 40506-0046, United States; 2 Pharmaceutical Sciences, University of Kentucky, Lexington, KY 40536-0596, United States

**Keywords:** acidic aqueous environments, carboxylic acids, electron microscopy, environmentally mediated dissolution, nanoceria

## Abstract

Cerium atoms on the surfaces of nanoceria (i.e., cerium oxide in the form of nanoparticles) can store or release oxygen, cycling between Ce^3+^ and Ce^4+^; therefore, they can cause or relieve oxidative stress within living systems. Nanoceria dissolution occurs in acidic environments. Nanoceria stabilization is a known problem even during its synthesis; in fact, a carboxylic acid, namely citric acid, is used in many synthesis protocols. Citric acid adsorbs onto nanoceria surfaces, limiting particle formation and creating stable dispersions with extended shelf life. To better understand factors influencing the fate of nanoceria, its dissolution and stabilization have been previously studied in vitro using acidic aqueous environments. Nanoceria agglomerated in the presence of some carboxylic acids over 30 weeks, and degraded in others, at pH 4.5 (i.e., the pH value in phagolysosomes). Plants release carboxylic acids, and cerium carboxylates are found in underground and aerial plant parts. To further test nanoceria stability, suspensions were exposed to light and dark conditions, simulating plant environments and biological systems. Light induced nanoceria agglomeration in the presence of some carboxylic acids. Nanoceria agglomeration did not occur in the dark in the presence of most carboxylic acids. Light initiates free radicals generated by ceria nanoparticles. Nanoceria completely dissolved in the presence of citric, malic, and isocitric acid when exposed to light, attributed to nanoceria dissolution, release of Ce^3+^ ions, and formation of cerium coordination complexes on the ceria nanoparticle surface that inhibit agglomeration. Key functional groups of carboxylic acids that prevented nanoceria agglomeration were identified. A long carbon chain backbone containing a carboxylic acid group geminal to a hydroxy group in addition to a second carboxylic acid group may optimally complex with nanoceria. The results provide mechanistic insight into the role of carboxylic acids in nanoceria dissolution and its fate in soils, plants, and biological systems.

## Introduction

### Background

Ceria nanomaterials have many applications, including acting as redox catalysts/metal supports [1], sunscreens [2], heat-resistant coatings [3], and much more [4]. Biomedical applications of ceria-based compounds as therapeutics have the potential to inhibit cancerous tumor growth [5], reduce radiation-induced damage [6], and heal wounds [7], among many other effects [8–9] cited in the introduction of [10]. Cerium atoms on nanoceria surfaces can store or release oxygen, cycling between Ce^3+^ and Ce^4+^; therefore, they can relieve oxidative stress within biological systems [11].

#### Nanoceria in plant systems

Nanoceria acts as colloids in aqueous environments, in the soil near plant root systems, and within bodily fluids. Acetic, citric, lactic, succinic, and tartaric acid secreted from plant roots are known to complex with metals/metal oxides within the rhizosphere [12–13]. Colloid stability of nanoceria is affected by temperature, pH, surface structure, surface-adsorbed organic and inorganic ligands, and metal/nonmetal ions and their concentrations in the solution surrounding the particles [14]. Nanoceria interacts with soil and plant roots, where it is known to dissolve and transform in the presence of chelating agents at low pH [15]. In cucumber plants, there is clear evidence of ceria uptake and transport throughout the plant. A fraction of the ceria formed cerium carboxyl complexes. No phytotoxicity was reported to the plant itself [16]. Nanoceria was partially biotransformed in cucumber plants to cerium phosphate within the roots and to cerium carboxylates in the shoots, presumably aided by carboxylic acids excreted by the roots [17]. Ceria transformation within cucumber plants was also found to be affected by phosphate, with a higher percentage of cerium carboxylates in the shoots in the absence of added phosphate [18]. Nanoceria can be taken up by food crops; however, limited biotransformation was observed in soil cultivated soybeans [19]. Coated and uncoated ceria nanoparticles were found in the roots and shoots of corn plants. The organic matter content of the soil also played a critical role in nanoceria uptake [20]. Cerium was detected in plant tissues, indicating nanoceria translocation within tomato plants [21]. The uptake and toxicity of nanoceria within radish seedlings were significantly reduced upon addition of a citric acid coating to the particle surface [22]. Nanoceria partially dissolved in the presence of organic acids in radish root exudates [23]. Nanoceria agglomeration was reported in algae growth medium beyond 28 h of exposure [24]. Collin et al. [25] urged future studies to look into environmental exposures and transformations of nanoceria surfaces.

#### Nanoceria within biological systems

Nanoceria has been shown to accumulate and persist in rats and mice for 90 days and five months [26]. A significant amount was present within liver, spleen, and bone marrow [27–28]. Yokel et al. [29] discussed the uptake, distribution, and toxicity of nanoceria within biological systems. Cellular uptake studies of nanoceria in lung adenocarcinoma (A549) cells favored particles with a negative zeta potential. However, positively charged particles resulted in greater bovine serum albumin adsorption. This suggests that surface interactions play a critical role in biological processes [30].

### Nanoceria dissolution

Nanoceria dissolution or agglomeration is pH-dependent and influenced by carboxylic acids. Nanoceria dissolution in aqueous solution was shown to be pH-dependent with dissolution occurring at pH values less than 7 [31]. Nanoceria (33 and 78 nm) dissolved at pH values less than 5 and to a greater extent at pH 1.65. The dissolution rate was proportional to the surface area [32]. Nanoceria dissolution was observed at pH 5.5 in the presence of citric acid and other reducing agents after 21 days [18]. Carboxylic acids accelerated nanoceria dissolution in aqueous acidic environments [33]. The dissolution rate, related to the nanoparticle surface area, was modeled to obtain dissolution rate coefficients [34]. Citrate-coated nanoceria particles were exposed to artificial lung, gastric, and intestinal fluids. The exposure resulted in loss or overcoating of the surface citrate, and, in some cases, agglomeration [10]. Exposure to vitamin C and glutathione led to dissolution-accompanied aggregation of mesoporous silica CeO_2_ nanoparticles [35]. The shortening of ceria nanorods from 25 to 8 nm after 14 days at 200 °C was observed in organic solvents [36]. Partially degraded nanoceria formed cerium phosphate within rats and mice, presumably by a dissolution/re-crystallization process [37–40]. In artificial soil solutions, partial dissolution was observed at pH 4, however, not at pH 7 or pH 9 over 28 days [41]. The molecular structural components of carboxylic acids mediating these effects have not been addressed.

In this study, nanoceria stability and dissolution in the presence of carboxylic acids was tested for up to 30 weeks. Sixteen carboxylic acids (Figure S1, Supporting Information File 1) were tested at pH 4.5, namely citric, malic, isocitric, glyceric, lactic, tartaric, α-hydroxybutyric, β-hydroxybutyric, succinic, pimelic, glutaric, tricarballylic, adipic, acetic, tartronic, and dihydroxymalonic acid. Controls, including ascorbic acid, ammonium nitrate, sodium nitrate, and water, were also tested. The goal was to test whether carboxylic acids stabilize or accelerate nanoceria dissolution in acidic aqueous environments and determine the mechanism of dissolution depending on the molecular structure of each ligand relating to agglomeration or stabilization. In addition, the influence of ambient laboratory light was studied to simulate exposure to sunlight compared to nanoceria suspensions protected from light.

### Light versus dark environments

To assess the effect of light, samples were placed on a laboratory bench near windows and compared to replicates stored in the dark to simulate plant environments and biological systems. Ceria has been considered a possible UV filter in sunscreens [2,42]. Oxygen defects in the crystal lattice of ceria can presumably be altered by UV irradiation causing a redox switching of the cerium atoms between Ce^3+^ and Ce^4+^. This could explain the observance of a blue shift of the absorption edge in the UVA region [43]. Studying the effects of UV irradiation on nanoceria would be informative for environmental applications. In biological systems, colloidal nanoceria dispersions were found to be non-toxic to fibroblasts and were capable of preventing damage from UV irradiation [44]. When exposed to artificial sunlight, ceria nanoparticles produced hydroxy radicals and induced lipid peroxidation of the gills of cardinal tetra, a native species of the Rio Negro region [45]. The citric acid coating can also be altered by UV irradiation. Photolysis of citric acid under a Hg lamp formed 2-methyl-2-hydroxysuccinic, 3-hydroxyglutaric, and tricarballylic acid, presumably due to CO_2_ and -OH release [46].

The Ce^3+^ to Ce^4+^ ratio on the surface of nanoceria has been shown to be altered by the addition of H_2_O_2_. Ceria nanoparticles with a high Ce^3+^/Ce^4+^ ratio transition from colorless to yellow upon addition of H_2_O_2_, due to oxidation of Ce^3+^ to Ce^4+^. The solution then transitions back to colorless after approximately 15 days, due to reduction of Ce^4+^ to Ce^3+^ [47–48]. A similar color change, from colorless to dark orange, was observed upon addition of H_2_O_2_ produced enzymatically from glucose oxidase, suggesting the use of this phenomenon as a colorimetric sensor for bioanalysis [49]. This color transition is an important observation since nanoceria degradation will likely result in the change in oxidation state of surface cerium ions from Ce^4+^ to Ce^3+^.

## Results and Discussion

### Nanoceria carboxylic acid dispersions

#### Color change

In the presence of light, the majority of the nanoceria dispersions changed color from light yellow to colorless (attributed to Ce^4+^ reduction to Ce^3+^) [47]. Nanoceria stored in the dark did not change color and remained light yellow. Dynamic light scattering (DLS) results showed that the particles exposed to light also degraded at a much greater rate than those that were protected from light.

**Figure 1:**
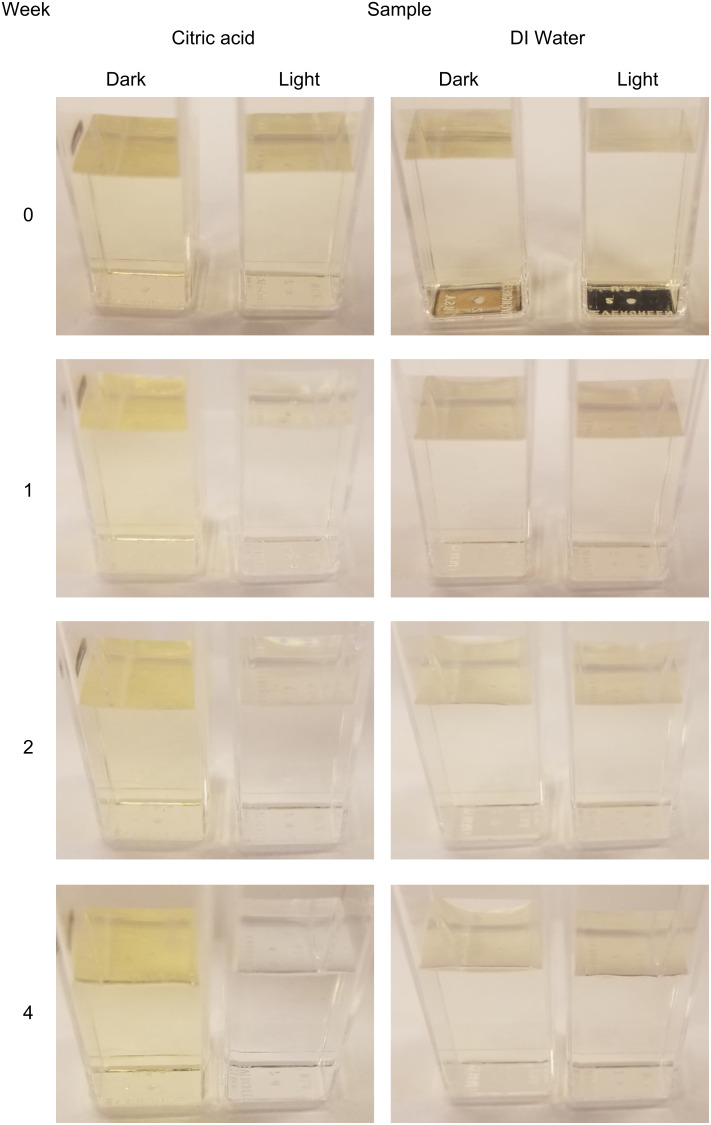
Nanoceria particles in citric acid (left column) or DI water (right column). The vials on the left of each panel were kept in the dark, while the vials on the right were exposed to UV radiation from sunlight. A reflection from the camera flash can be observed in the upper right panel that is not present in the other samples.

Nanoceria exposed to citric acid or DI water under light and dark conditions up to four weeks are shown in Figure 1. The vial on the left was kept in the dark, while the vial on the right was exposed to light. The sample in the citric acid column exposed to light for four weeks showed a color change from yellow to clear, possibly indicating a valence state change from Ce^4+^ to Ce^3+^. The citric acid sample exposed to light appears to be completely colorless after four weeks. No color change was present for the sample kept in the dark. A slight color change was noticeable for the control sample in water; however, a yellow tint was still present after four weeks, for both samples, exposed to dark and light.

The color change from yellow to colorless could also be representative of particle dissolution, due to the valence state change from Ce^4+^ to Ce^3+^ during dissolution. The particles in citric acid exposed to light were completely dissolved after four weeks as indicated by DLS and transmission electron microscopy (TEM), shown in Table 1 and Table 2, respectively. In Table 2 of Grulke et al. [34], changes in the valence state at the edge and core of the nanoparticles were shown over twelve weeks of dissolution. The Ce^3+^ concentration increased as the particle size decreased, but only slightly. Figure 7 of Yokel et al. [33] shows little to no change within four weeks; however, after twelve weeks, an increase in the electron energy loss spectroscopy M5 peak, corresponding to an increase in the Ce^3+^ valence state, is apparent.

**Table 1:** DLS results for nanoceria grouped by nanoceria stabilization/dissolution category. Each 3D graph contains exposure time, in h, on the *x* axis; hydrodynamic diameter, in nm, on the *y* axis; and peak height/size percentage on the *z* axis. The bars are representative of the percentage of the samples at that size. Most conditions yielded bimodal size distributions for which the smaller size is given in blue, and the larger size is given in red. The *y* axis shifts between linear and logarithmic coordinates to best show the hydrodynamic particle size before and after agglomeration.

	Conditions	
	Light	Dark

Group 1		

Citric acid	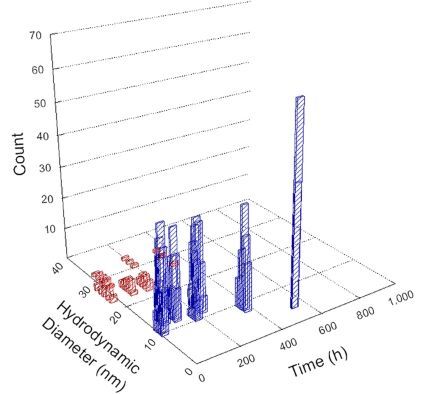	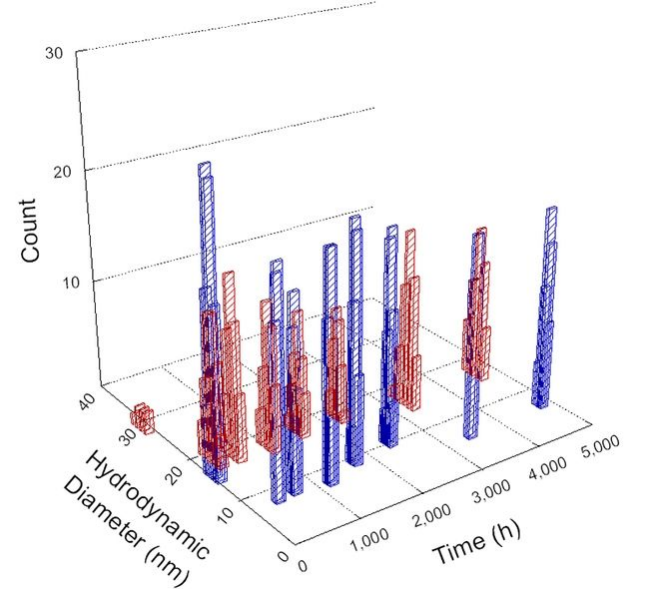
Malic acid	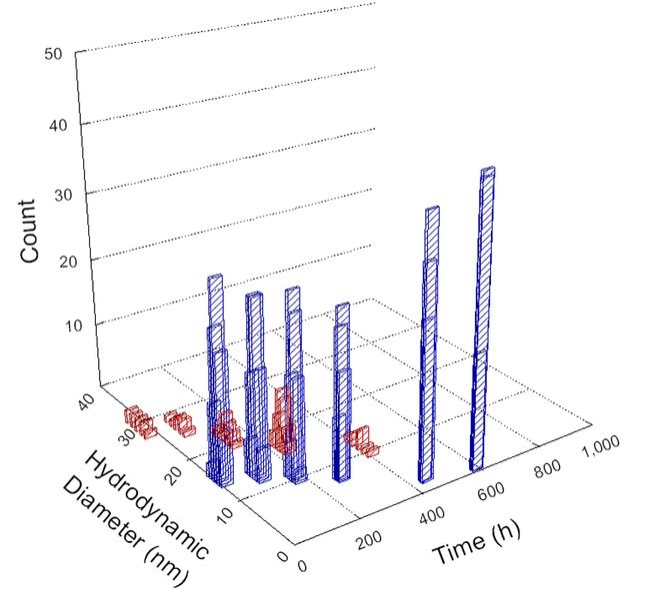	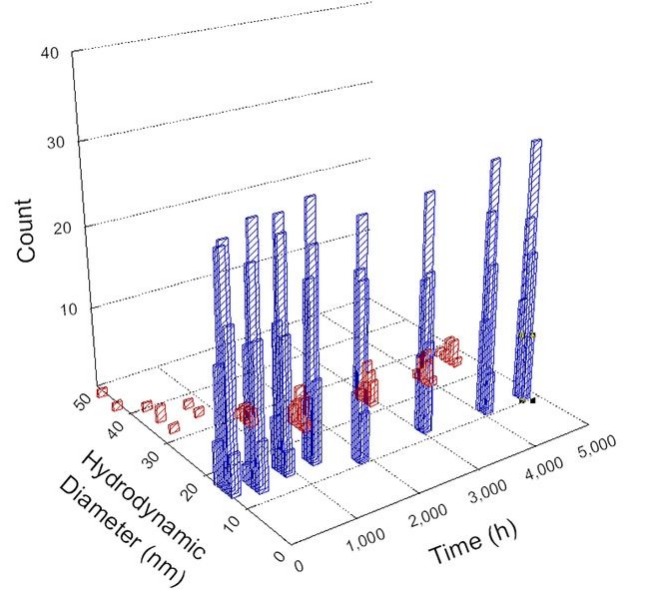
Isocitric acid	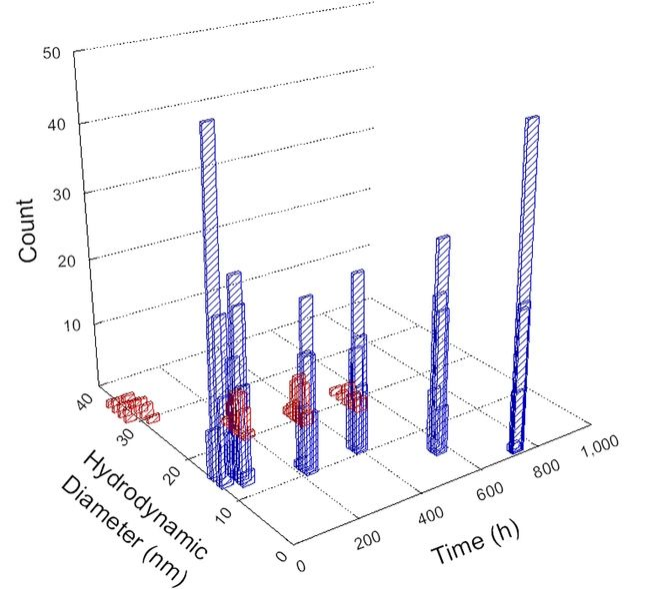	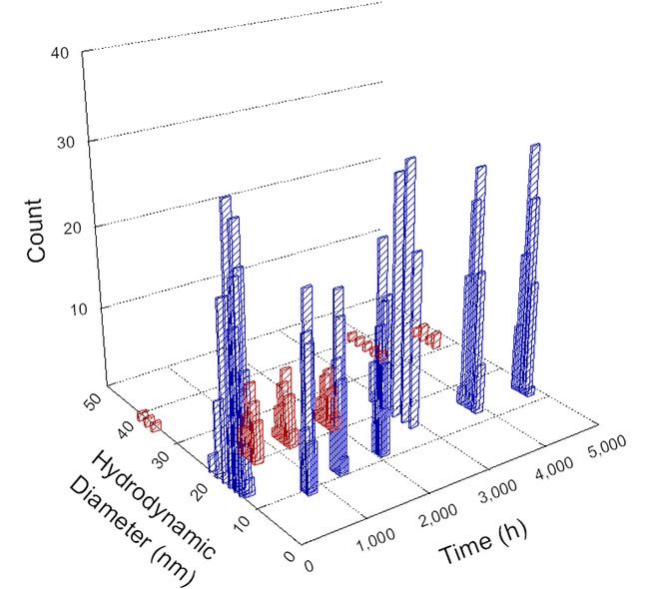

Group 2		

Glyceric acid	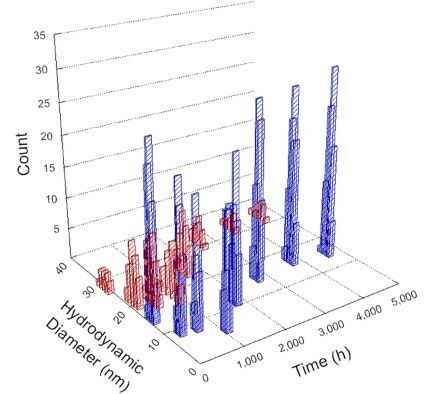	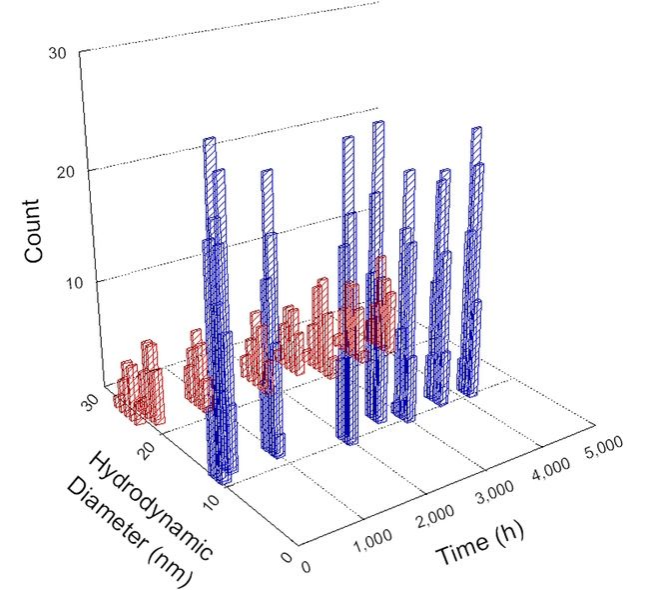

Group 3		

Lactic acid	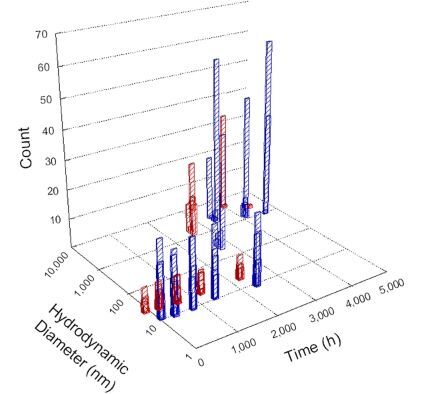	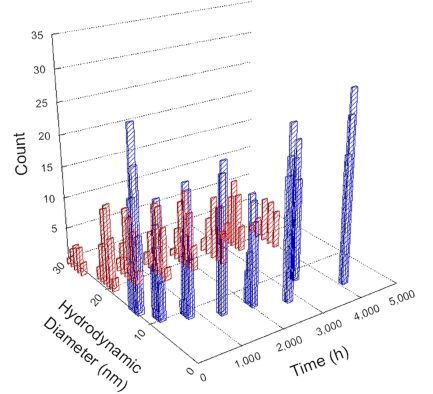
Tartaric acid	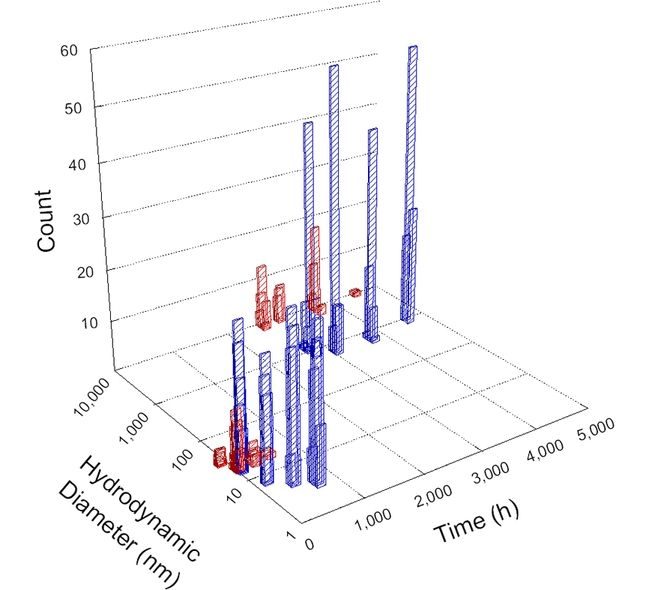	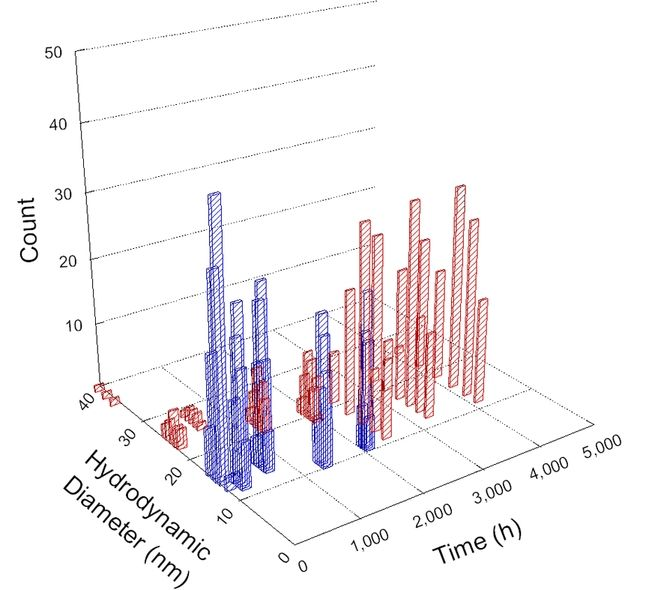
NH_4_NO_3_	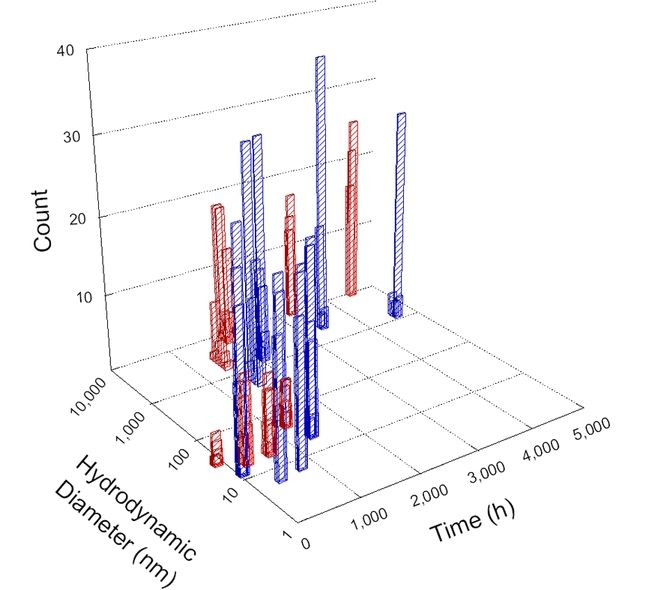	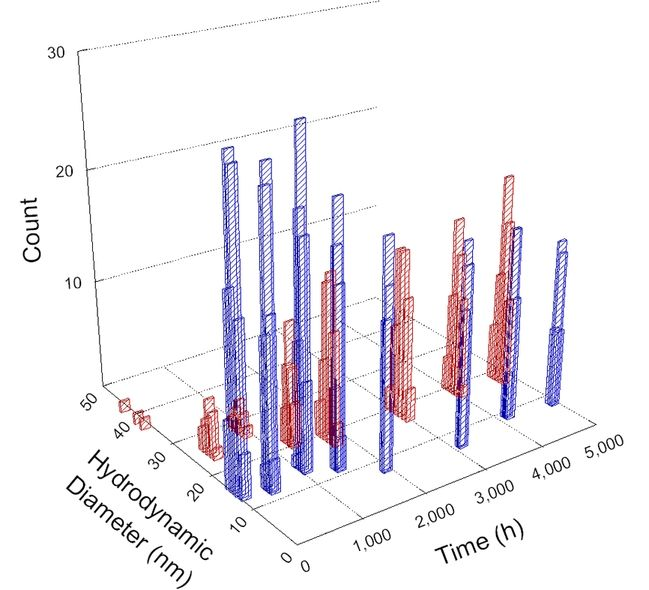
Water	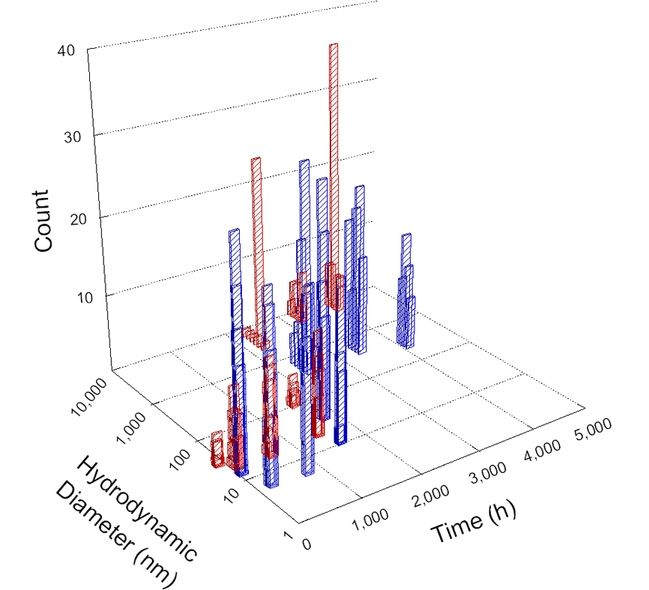	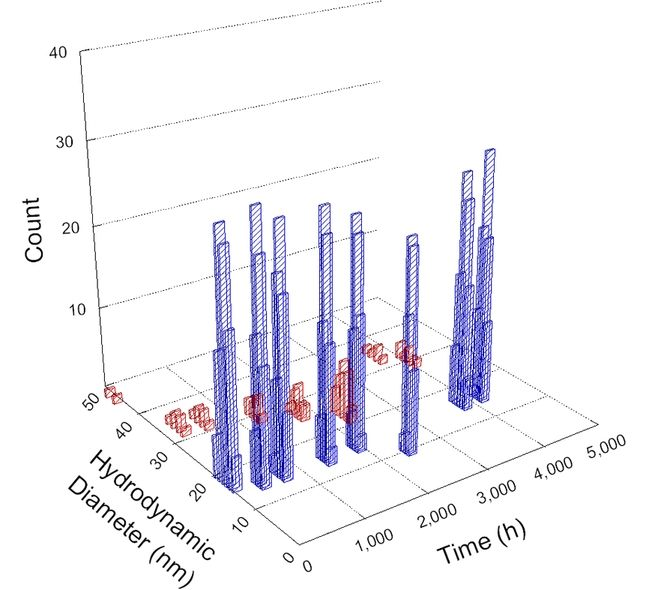

Group 4		

α-Hydroxybutyric acid	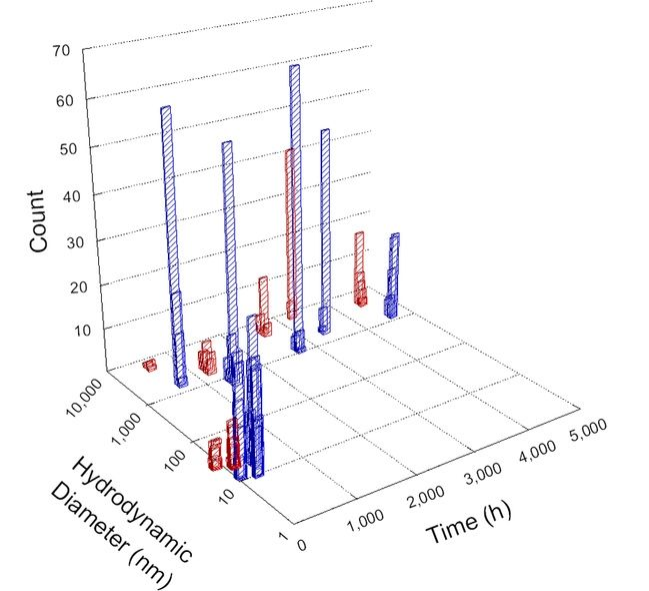	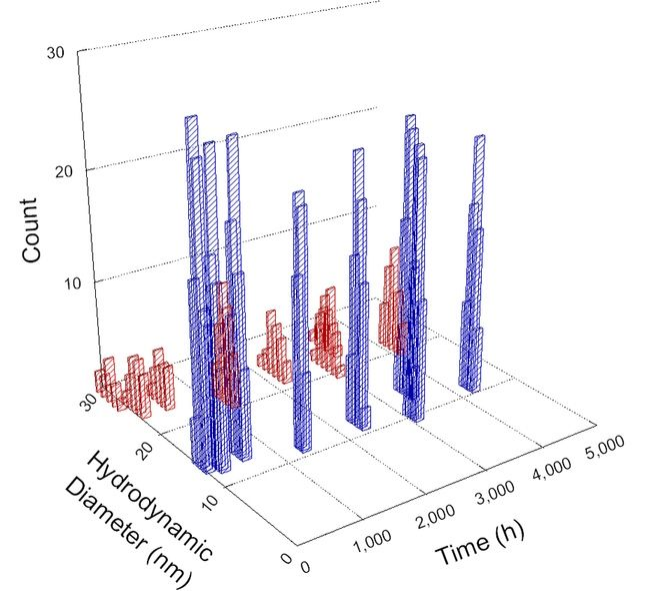
β-Hydroxybutyric acid	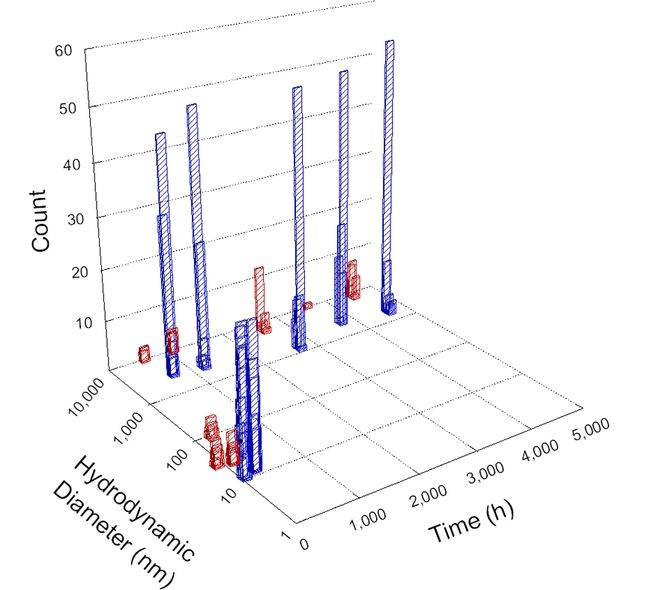	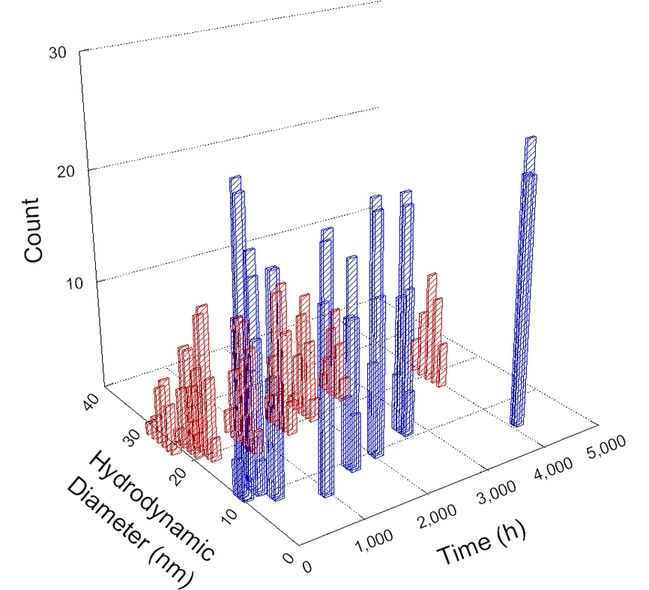
Succinic acid	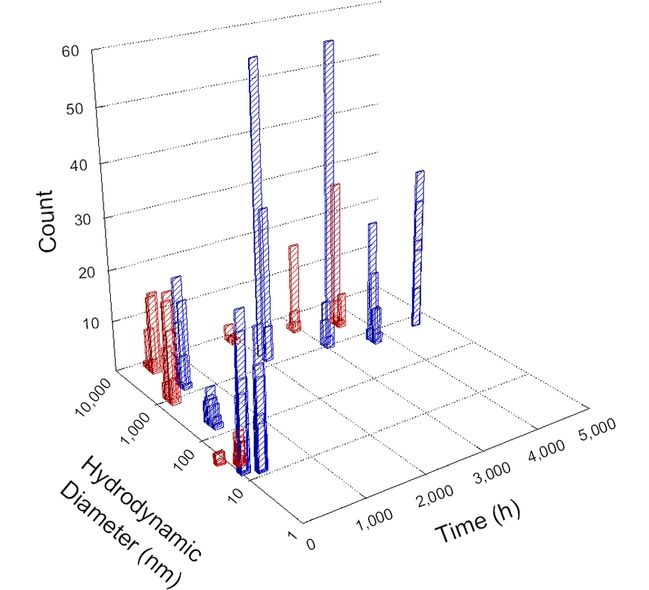	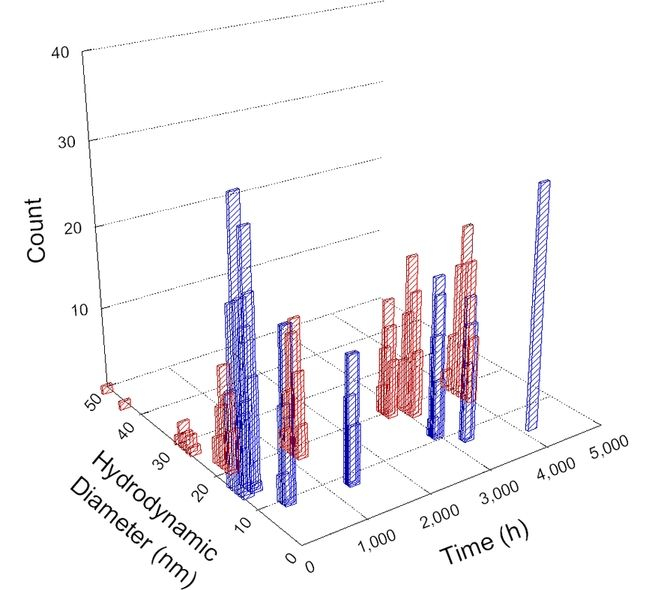
Pimelic acid	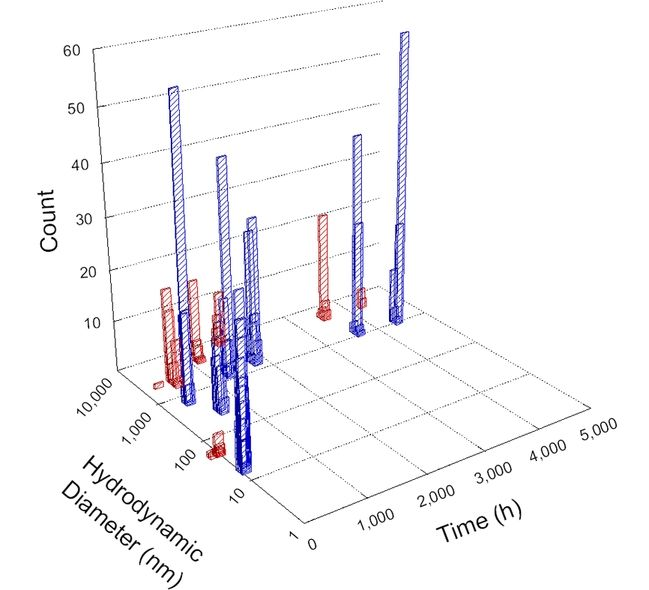	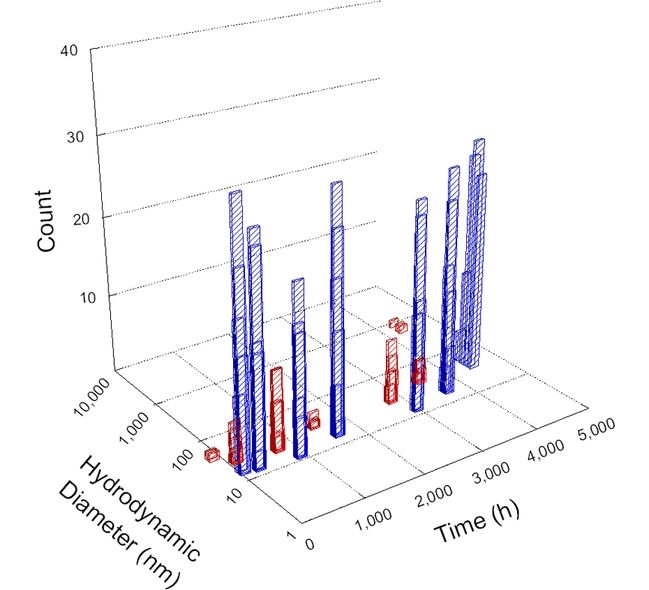
Glutaric acid	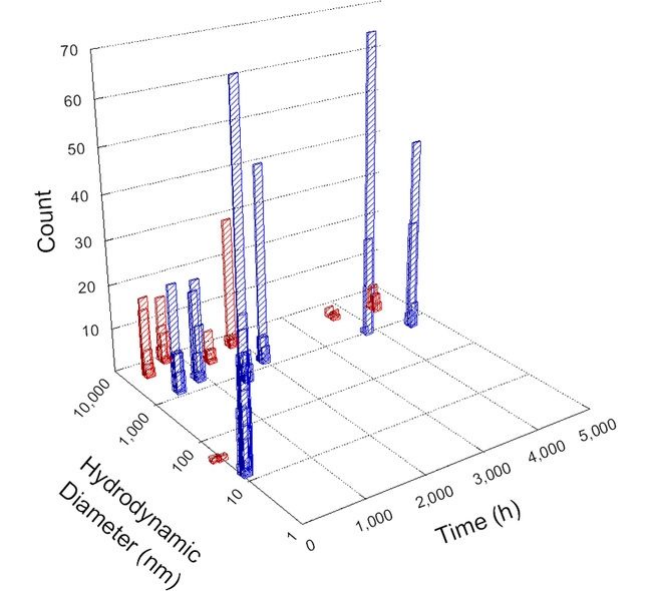	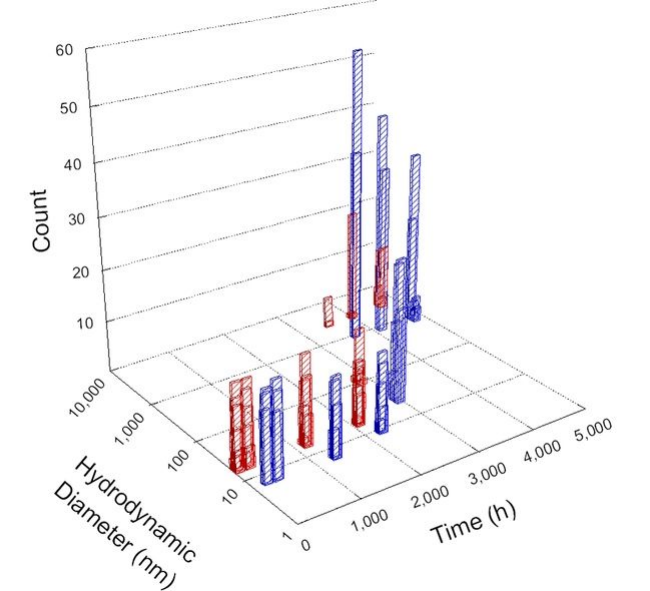
Tricarballylic acid	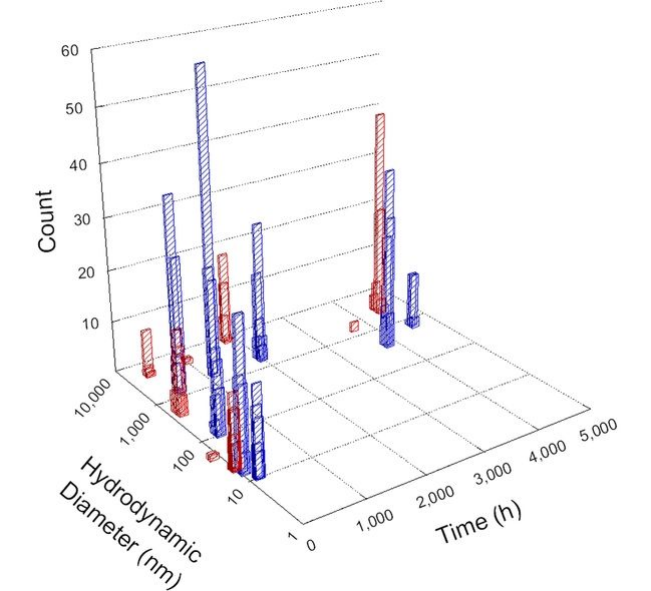	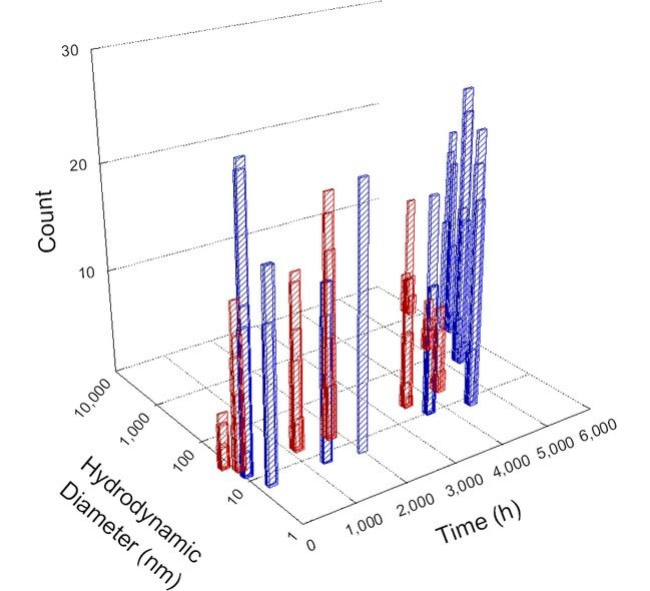
Adipic acid	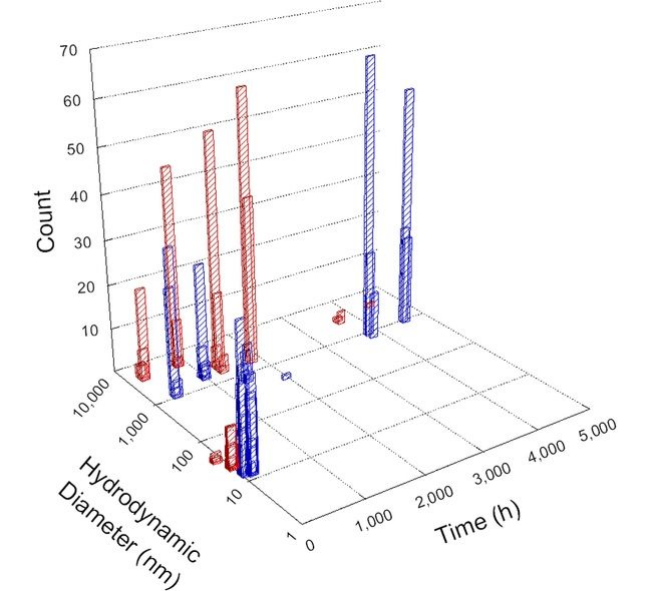	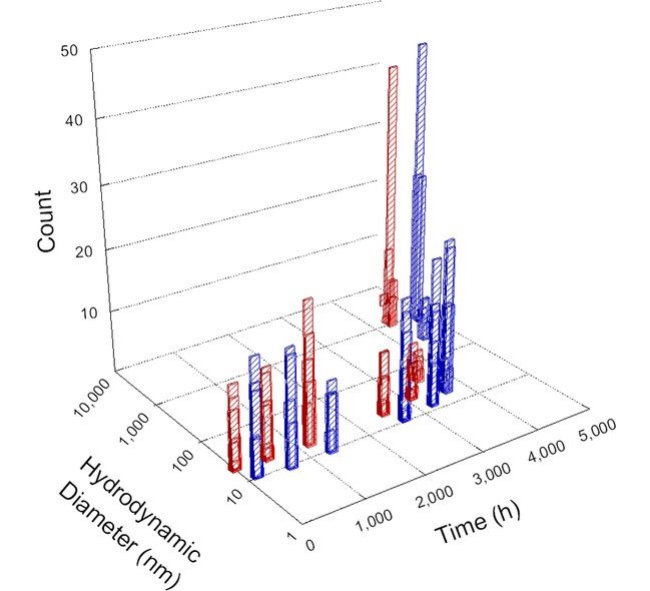
Acetic acid	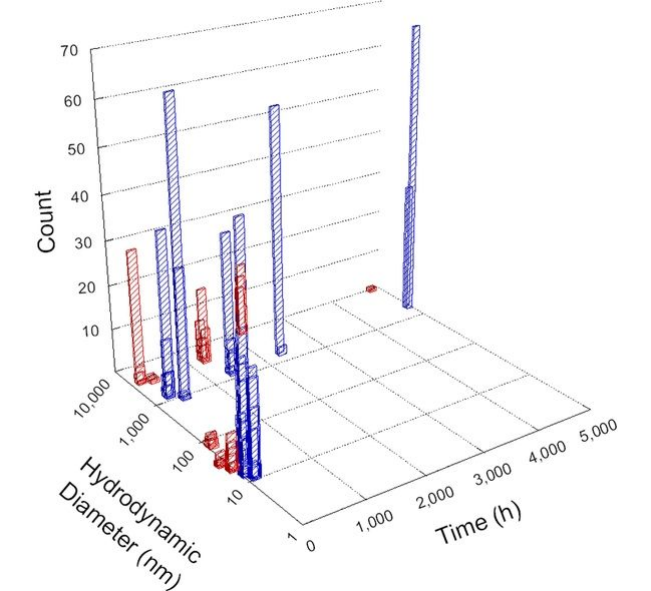	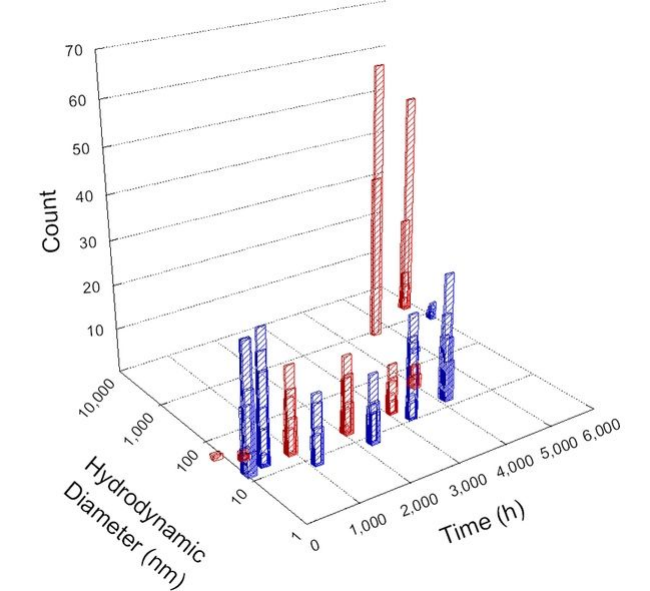
NaNO_3_	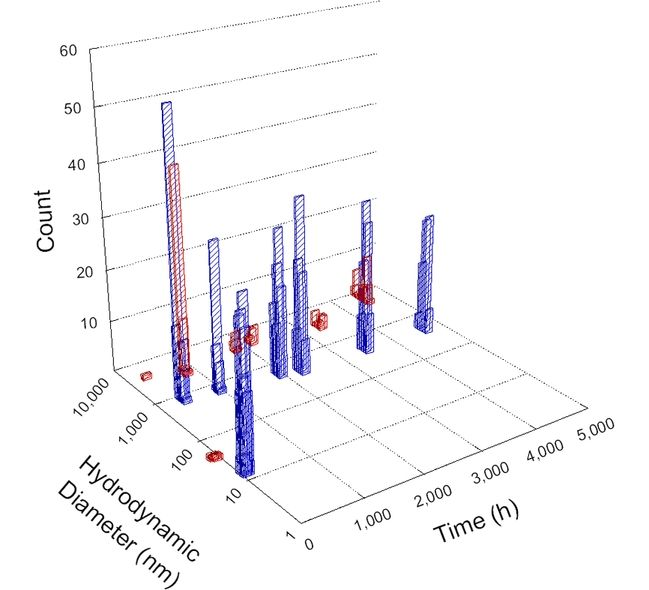	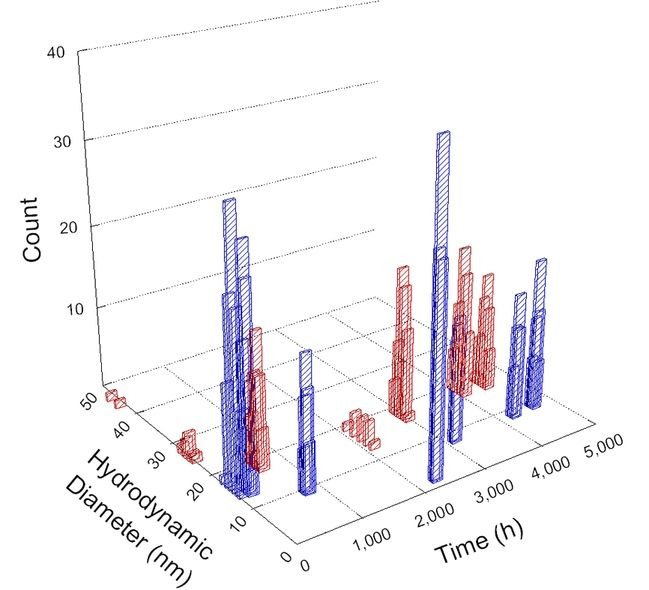

Group 5		

Tartronic acid	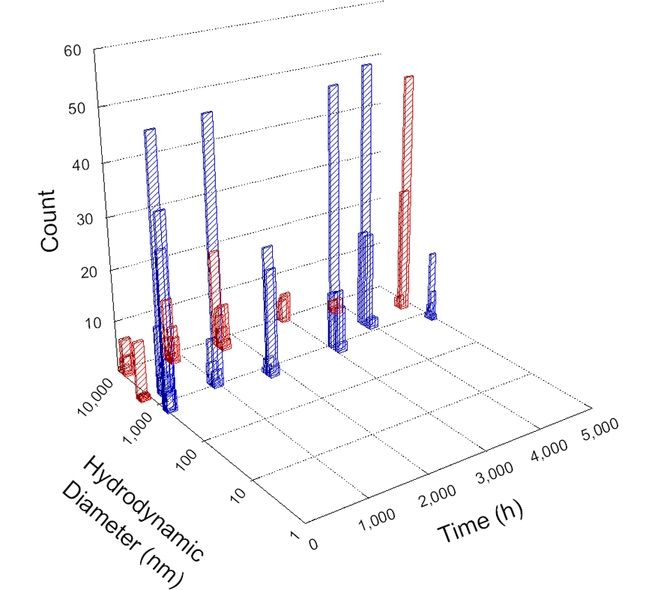	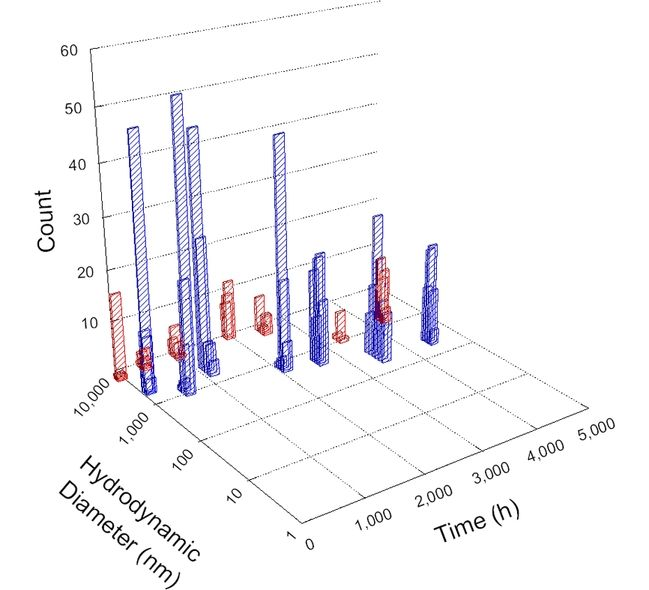
Dihydroxymalonic acid	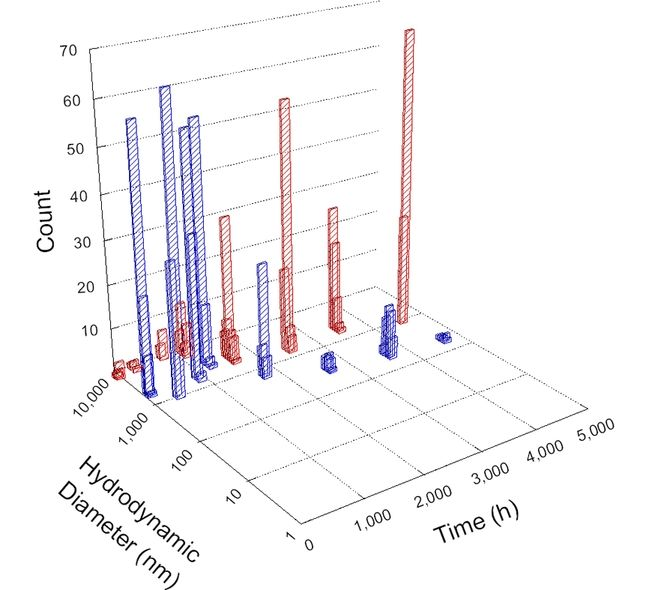	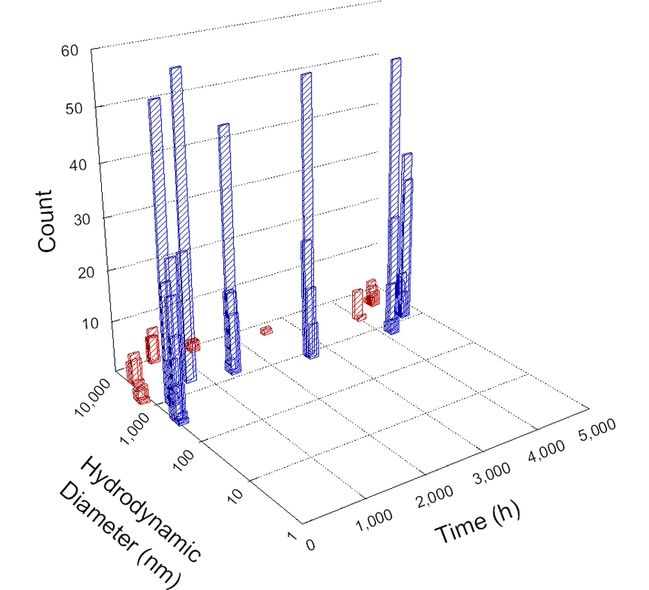
Ascorbic acid	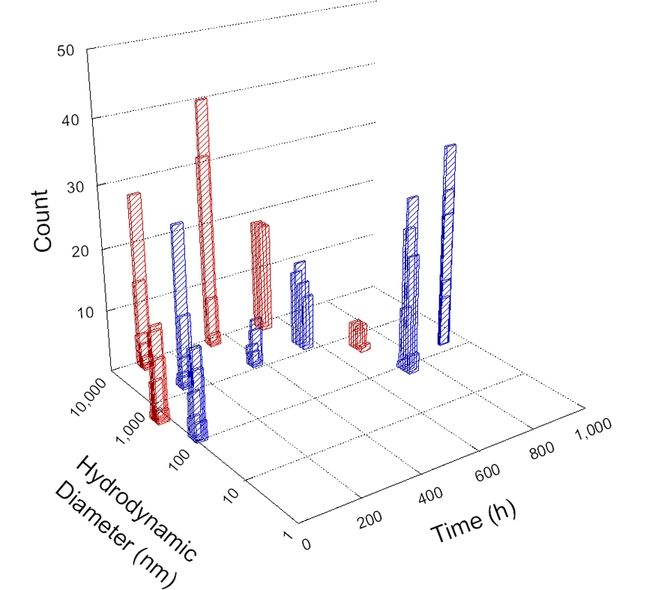	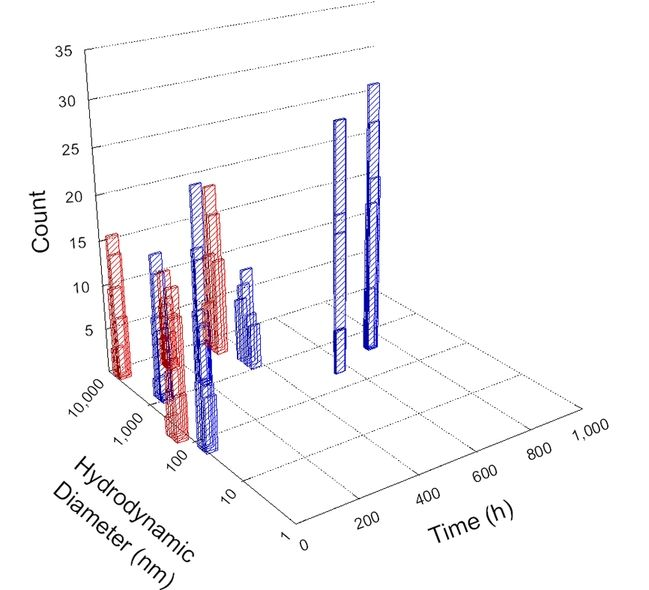

**Table 2:** TEM images at increasing magnification for nanoceria exposed to citric and β-hydroxybutyric acid in sunlight and stored in the dark. The images were obtained at 150k, 500k, and 1050k magnification for citric acid exposed nanoceria and 58k, 150k, and 500k magnification for ß-hydroxybutyric exposed nanoceria. The scale bars for the citric acid images are 50, 20 and 10 nm, respectively, and for the ß-hydroxybutyric images are 200, 50 and 20 nm, respectively.

	Week			

Citric acid (light)	0	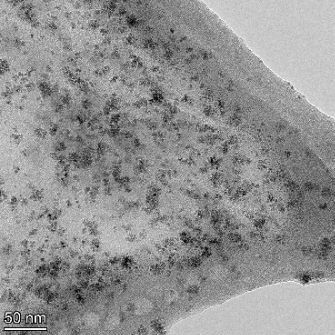	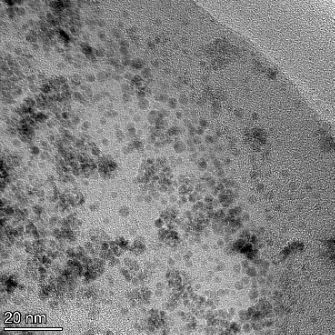	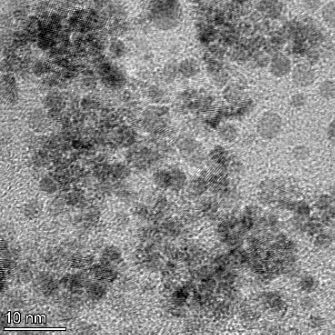
	1	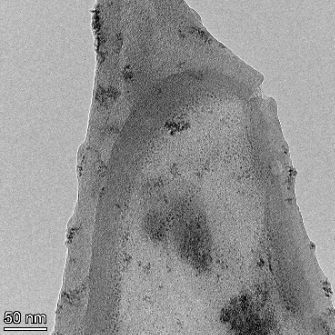	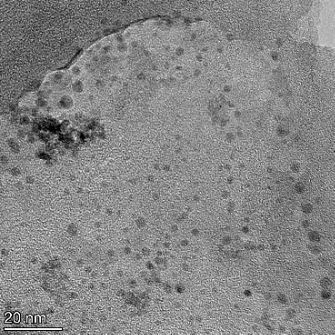	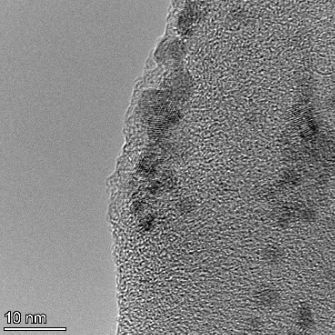
	2	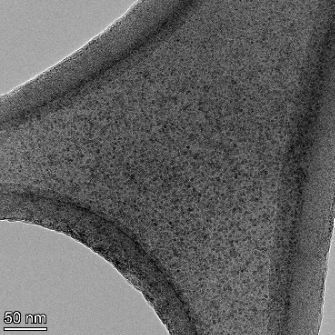	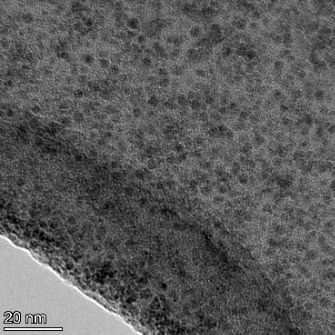	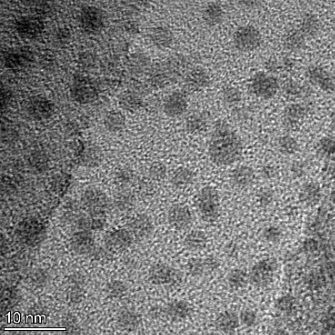
	4	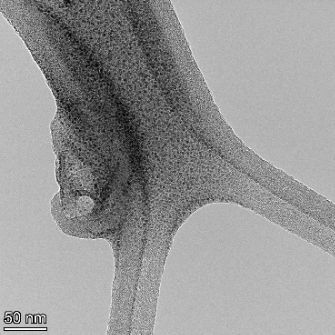	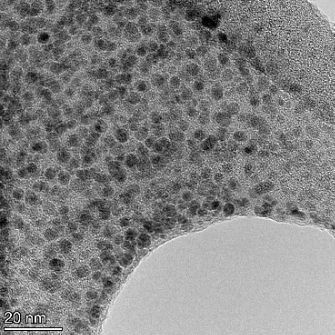	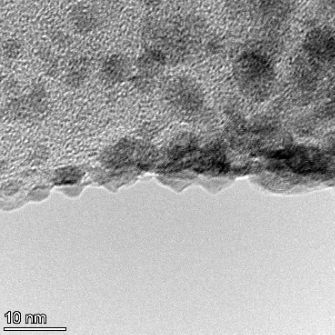
	8	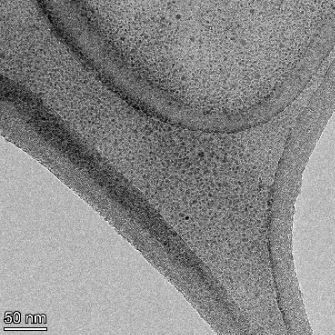	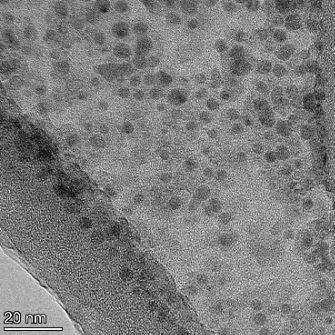	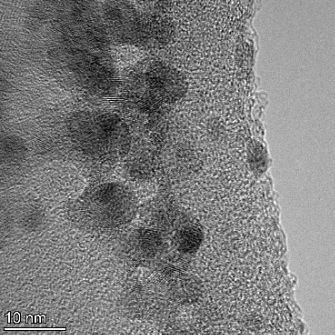

	Week			

Citric acid (dark)	0	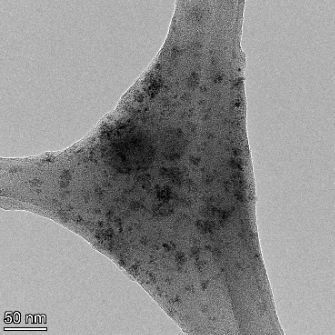	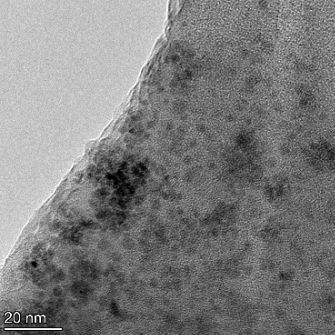	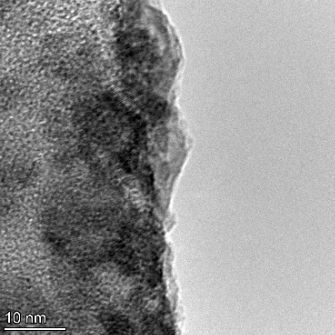
	1	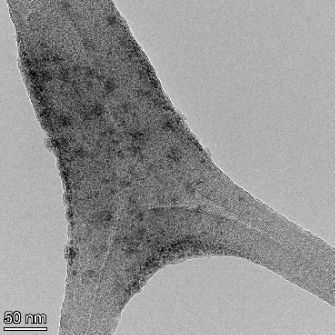	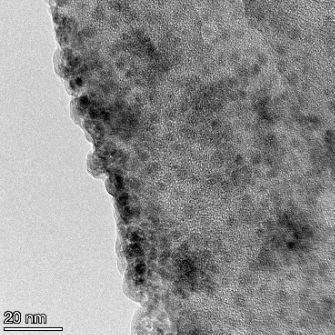	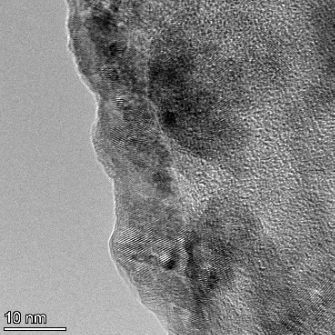
	2	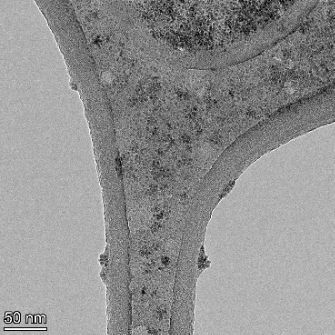	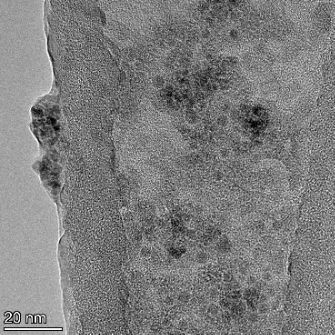	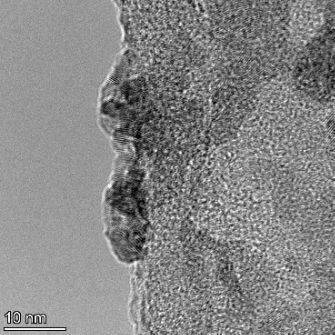
	4	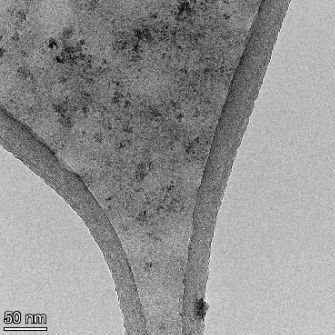	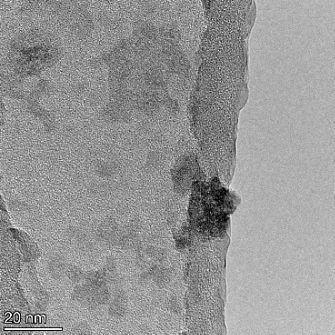	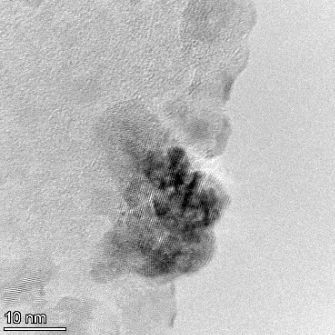
	8	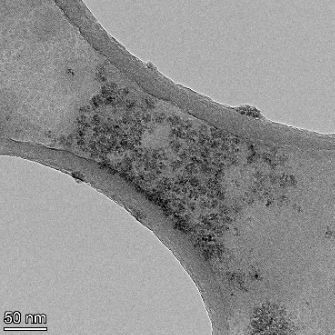	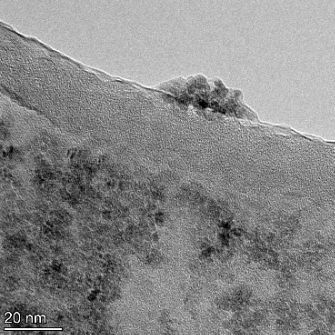	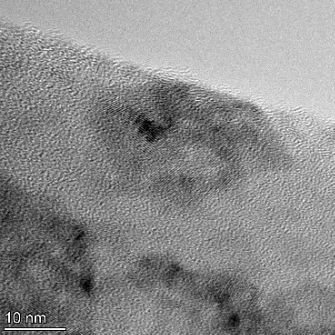

	Week			

β-Hydroxybutyric acid (light)	0	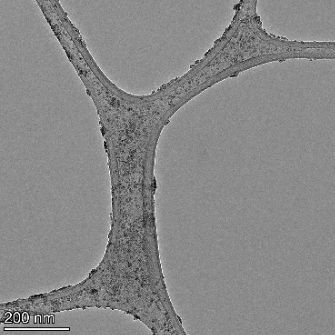	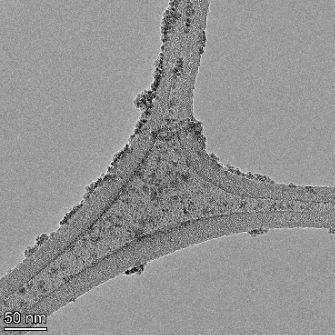	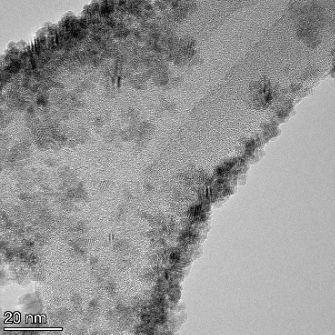
	1	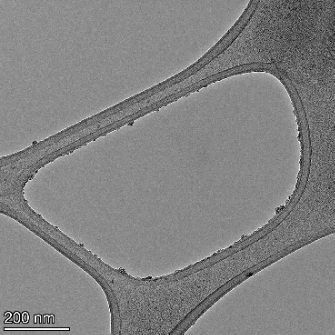	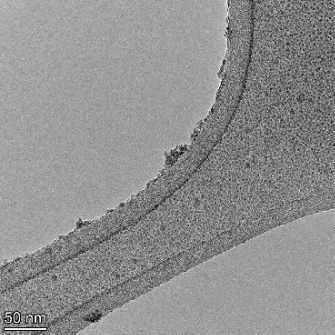	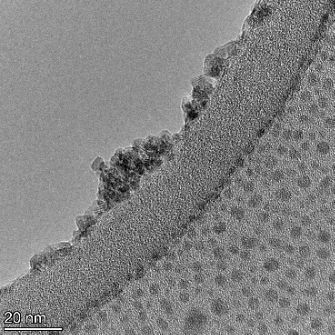
	2	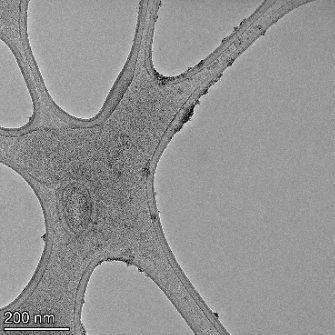	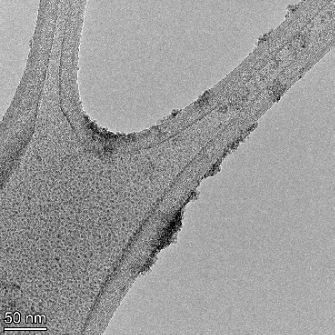	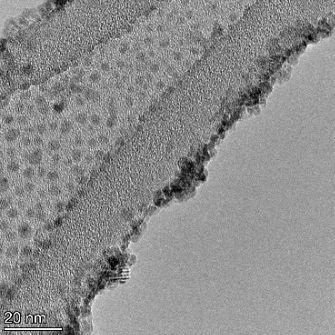
	4	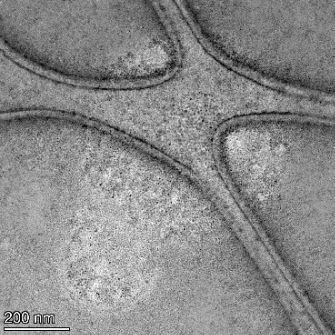	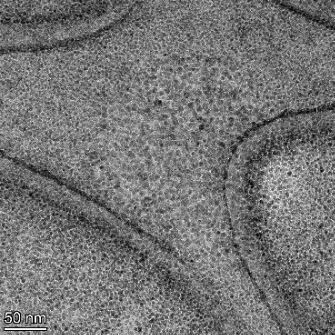	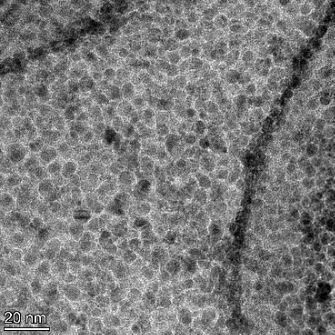
	8	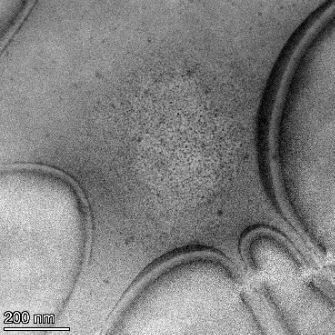	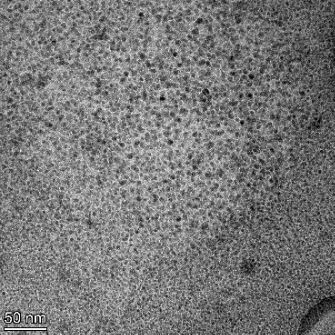	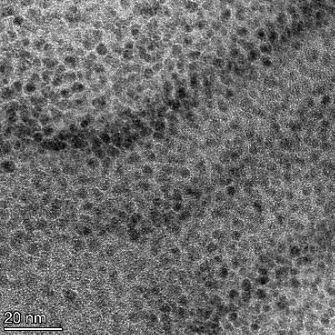

	Week			

β-Hydroxybutyric acid (dark)	0	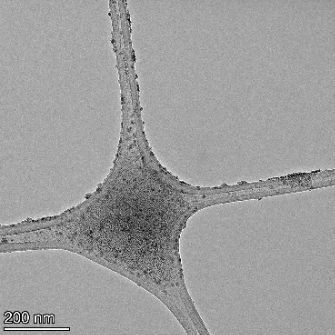	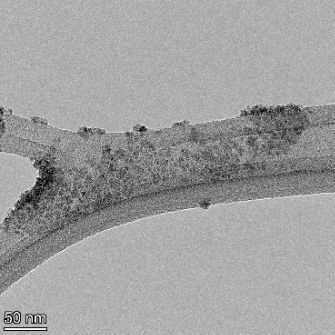	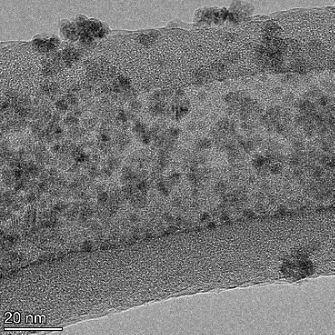
	1	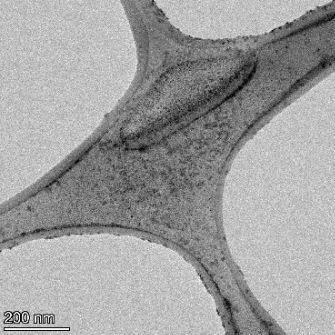	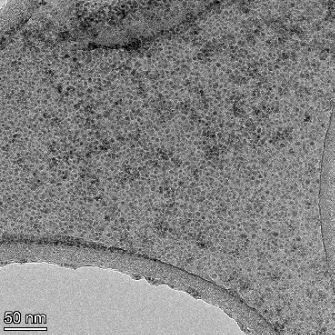	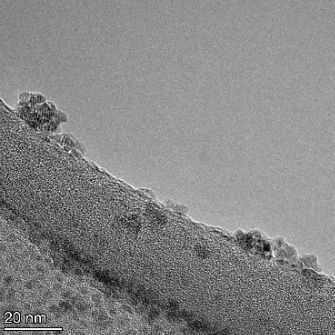
	2	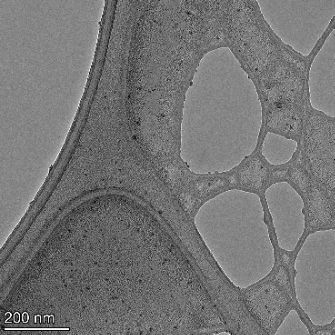	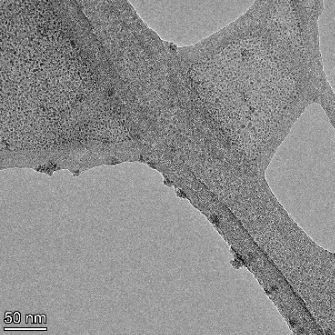	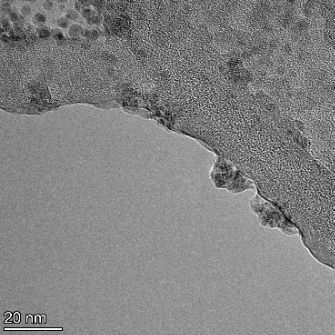
	4	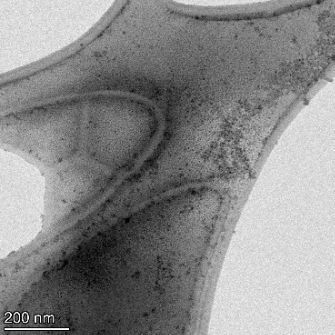	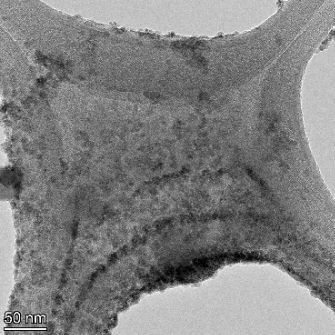	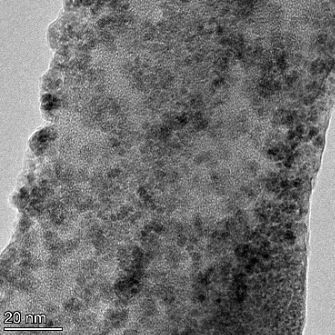
	8	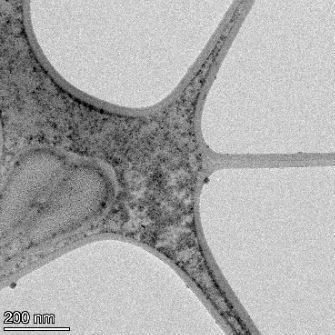	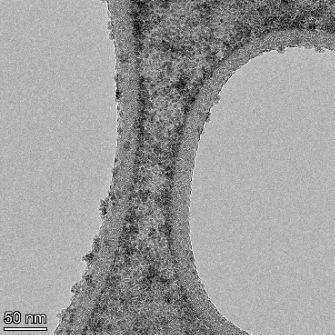	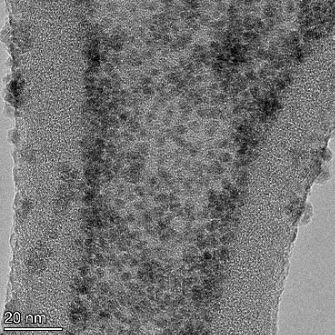

#### DLS analysis

DLS results of the 20 chemicals studied under the two conditions are shown in Table 1. Numerical values of these results are given in Table S1 (Supporting Information File 1). Each sample typically contained a bimodal distribution, represented by the blue and red bars on the 3D graphs. The peak heights of these bars represent the sample percentage at that size. In some samples, nanoceria completely dissolved before the end of the experiment; those experiments were terminated at 1000 h. The average bimodal size distribution for the nanoceria suspension at time zero among all conditions was 13.8 and 33.6 nm with a standard deviation of 2.1 and 8.0 nm, respectively.

The results shown in Table 1 can be summarized in five categories. The first contains three carboxylic acids in which the nanoceria completely dissolved within 1000 h when exposed to light. The second contains one carboxylic acid that prevented agglomeration over the entire experimental duration but did not completely dissolve the nanoceria particles. The third contains four chemicals that prevented agglomeration for an extended time (approximately 1500–2000 h). The fourth contains nine chemicals in which nanoceria showed initial dissolution and, subsequently, agglomeration within 300–400 h, when exposed to light. The fifth contains three carboxylic acids in which nanoceria agglomerated immediately, whether exposed to light or not. All categories, expect for group five, prevented agglomeration in the dark for most of the experimental duration. Five samples in group four agglomerated in the dark towards the end of the experiment (after approximately 4000–5000 h). The following identifies the members of the five groups and describes their effect on nanoceria dissolution/stability.

Group one includes nanoceria exposed to citric, malic, and isocitric acid. For all three samples stored in the dark, there was an immediate reduction in hydrodynamic particle size, followed by slow dissolution over time. The solution color remained yellow for the experimental duration. For the samples exposed to light, there was an immediate reduction in hydrodynamic particle size accompanied by a color change from yellow to clear. The nanoceria particles decreased to 8.4, 7.3, and 10.9 nm in diameter within 250 h for citric, malic, and isocitric acid, respectively (shown in Table S1, Supporting Information File 1). They fully dissolved within 600 h for citric and malic acid samples, and 800 h for isocitric acid. Light exposure may have contributed to the acceleration of nanoceria dissolution, since nanoceria particles are known to be photoreduced upon UV radiation exposure, demonstrated by UV exposure of 2 and 9 nm citrate-coated ceria nanoparticles in aqueous solution at pH 6.5 to 7.0 [50] and 3 nm ceria nanoparticles in aqueous solution at pH 2 [51]. Furthermore, this result provides insight into the molecular structure of the ligand needed to stabilize the nanoceria surface and to prevent agglomeration. All three acids contain a carboxylic acid functional group geminal to a hydroxy group. NMR results confirmed that this type of structure may be influential in bonding with ceria [52].

Group two includes only one sample, namely glyceric acid. When stored in the dark, there was a slight reduction in hydrodynamic particle size over time, and the color remained yellow, similar to the response to group one. However, when exposed to light, there was an immediate reduction in hydrodynamic particle size to 4.9 nm in diameter for about 1000 h, then a steady increase to 14.5 nm for the remainder of the time. Also, a color change from yellow to clear, then back to yellow just before the increase in size was observed. All hydrodynamic particle sizes were less than 50 nm; therefore, there was no evidence of significant particle agglomeration.

Group three contains lactic and tartaric acid, ammonium nitrate, and water. Again, there was a small reduction in hydrodynamic particle size over time and no noticeable color change when stored in the dark, similar to groups one and two. The overall decrease in hydrodynamic particle size in the presence of lactic acid was associated with the highest dissolution rate across all samples stored in the dark, consistent with results from prior studies [33–34]. When exposed to light, all samples showed a small reduction in hydrodynamic particle size over approximately 1500–2000 h, followed by agglomeration to micrometer-sized particles. Also, the color changed from yellow to clear, then back to yellow just before agglomeration, similar to group two.

Group four includes nine samples, that is, α-hydroxybutyric, β-hydroxybutyric, succinic, pimelic, glutaric, tricarballylic, adipic, and acetic acid plus sodium nitrate. As was the case for groups one through three for the samples stored in the dark, there was a small reduction in hydrodynamic particle size over time with no noticeable change in color. Nanoceria agglomerated in the presence of succinic, glutaric, adipic, and acetic acid towards the end of the experiment, between 4000 and 5000 h. For those exposed to light, there was a slight reduction in size, followed by agglomeration around 300–400 h. The color remained yellow throughout the experimental duration. The majority of these samples agglomerated at drastically different time points under light or dark conditions. This again is consistent with the hypothesis that UV light accelerates nanoceria dissolution due to the photoactive nature of nanoceria upon exposure to UV radiation.

Group five contains three samples, namely tartronic, dihydroxymalonic, and ascorbic acid. Nanoceria particles immediately agglomerated when exposed to these acids, in both light and dark environments. This shows that these acids were unable to create a stable environment for the nanoceria particles. The color remained yellow throughout the experimental duration when exposed to tartronic and dihydroxymalonic acid. However, a color change from yellow to reddish-brown was observed for the nanoceria in contact with ascorbic acid, as previously observed [53]. Nanoceria agglomerates completely dissolved in ascorbic acid within 1000 h. Ascorbic acid dissolution-accompanied aggregation of nanoceria was also shown by Muhammad and co-workers [35].

#### TEM analysis

TEM analysis was conducted on two samples, citric and β-hydroxybutyric acid, to compare to the DLS results. They were chosen to represent samples from groups one and four. In the presence of light, nanoceria exposed to citric acid did not agglomerate and completely dissolved within two weeks. In contrast, particles exposed to β-hydroxybutyric acid agglomerated within four weeks. Shown in Table 2 are TEM images at varying magnifications of each sample exposed to light or stored in the dark. Nanoceria particles exposed to both acids in the dark appear very similar, as do the DLS results. Nanoceria was present in each sample in 15–25 nm agglomerates throughout eight weeks. Large micrometer-sized agglomerates were not detected by TEM under dark conditions for either sample.

However, the samples exposed to light produced drastically different TEM images, as expected from the DLS results. The number of nanoceria particles exposed to citric acid was reduced between weeks 0 and 1. By week 2, most of the particles completely disappeared with no evidence of cerium determined by energy-dispersive X-ray spectroscopy (EDS). Instead, approximately 5 nm features were observed at weeks 2, 4, and 8. These features were not stable and were altered by the high-intensity electron beam, confirming that they are not cerium oxides (Figure S2, Supporting Information File 1). Allen et al. [54] showed similar results and considered how these fine features may be associated with a complex of cerium and hexamethylenetetramine. However, as evident from the STEM EDS mapping analysis (Figure S3, Supporting Information File 1), the features include sodium, likely from sodium nitrate. From these results, it also appears that the 15–25 nm agglomerates on the edges of the lacey carbon film are nanoceria.

The presence of β-hydroxybutyric acid appeared to reduce the overall number of nanoceria particles from week 0 through week 2, similar to citric acid. However, by week 4, large micrometer-sized agglomerates were detected, made up of hundreds of nanoceria particles that persisted throughout the experimental duration. An EDS scan of the agglomerate confirmed the presence of cerium (Figure S4, Supporting Information File 1). This confirms the DLS results of this sample at four weeks (Table 1) that ceria agglomerated into micrometer-sized particles.

#### Functional group analysis

As mentioned earlier, the carboxylic acid group geminal to a hydroxy group may be essential for nanoceria–ligand complexation. Nine of the tested carboxylic acids contain this structure. Three of them (citric, malic, and isocitric acid) completely dissolved nanoceria when exposed to light and one (glyceric acid) prevented agglomeration throughout the entire experimental duration. Two acids (lactic and tartaric acid) stabilized nanoceria for an extended time before agglomeration. One acid (α-hydroxybutyric acid) prevented agglomeration for a short time, while the remaining two (tartronic and dihydroxymalonic acid) led to immediate agglomeration upon exposure. This result indicates that while the carboxylic acid group geminal to a hydroxy group may be important, other functional groups may also play a role in stabilizing citrate-coated nanoceria. For instance, citric, malic, and isocitric acid all contain at least one more carboxylic acid group (citric and isocitric acid contain two additional groups). The same is true for tartaric, tartronic, and dihydroxymalonic acid; however, the carbon chain is shorter for these molecules and does not contain any CH_2_ groups, which may prevent molecular configurations required for nanoceria complexation. The remaining three acids (glyceric, lactic, and α-hydroxybutyric acid) do not contain a second carboxylic acid group. Furthermore, citric, malic, and isocitric acid molecules all have higher ratios of carboxylic acid and hydroxy groups to aliphatic carbon atoms (Table S2, Supporting Information File 1). Overall, the results suggest that a long carbon chain backbone containing a carboxylic acid geminal to a hydroxy group in addition to a second carboxylic acid group may be necessary for nanoceria complexation.

## Conclusion

Citric acid-coated nanoceria was exposed to 16 carboxylic acids, ascorbic acid, sodium nitrate, ammonium nitrate, and water at pH 4.5 in light and dark environments to simulate sunlight exposure to plants and within biological systems. The hydrodynamic particle size was determined by DLS over 30 weeks. For most light exposure conditions, a color change from yellow to colorless was observed, indicating a valence state transition from Ce^4+^ to Ce^3+^. As nanoceria particles dissolve and become smaller, the surface oxidation state transitions from Ce^4+^ to Ce^3+^. Of the 16 carboxylic acids tested, nanoceria exposed to only three acids (citric, malic, and isocitric acid) fully dissolved in less than 1000 h when exposed to light. Glyceric acid prevented agglomeration, but nanoparticles were still present after 30 weeks of exposure. Nanoceria formed micrometer-sized particles in the remainder of the samples either immediately or between 300 and 2000 h of exposure. In the dark, particles were still present at the end of 30 weeks for all carboxylic acids and the controls. This indicates that light accelerates nanoceria dissolution and stabilizes some nanoceria–carboxylic acid complexes. Lactic acid dissolved the particles at a greater rate than any other acid in the dark. TEM imaging of nanoceria in citric and β-hydroxybutyric acid supported the DLS results. Relating the carboxylic acid composition (number of carboxylic acid groups, total number of carbons, aliphatic carbons, and hydroxy groups and their molecular ratios) to the nanoceria dissolution rate provides insight into the molecular components of carboxylic acids that affect nanoceria dissolution or stable complexation. A long carbon chain backbone containing a carboxylic acid group geminal to a hydroxy group in addition to a second carboxylic acid group may optimally form complexes with nanoceria. The results provide mechanistic insight into the role of carboxylic acids in nanoceria dissolution and the fate of nanoceria in soils and plants. They suggest that lactic and other carboxylic acids accelerate nanoceria dissolution in the soil, releasing Ce^3+^, some of which is taken up by plant roots, is complexed to carboxylic acids, and persists as cerium carboxylates in aerial plant structures. Further determination of the cerium species in aerial plant structures is needed to determine if the cerium carboxylates are soluble/stable complexes, as predicted for cerium citrate, or precipitates, as suggested by the cerium carboxylate complexes in groups three to five above.

## Experimental

### Materials

The following chemicals, including their sources, purity, and CAS numbers, were used: citric acid monohydrate, Fisher, ACS grade, 5949-29-1; ᴅʟ-malic acid, Alfa Aesar, 98%, 6915-15-7; ᴅʟ-isocitric acid, trisodium salt hydrate, Acros Organics, 95%, 1637-73-6; ᴅʟ-lactic acid, TCI, >85%, 50-21-5; ᴅʟ-glyceric acid, TCI, 20% in water (ca. 2 mol/L), 473-81-4; ᴅʟ-tartaric acid, TCI, >99%, 133-37-9; glutaric acid, Acros Organics, 99% 110-94-1; tricarballylic acid, Alfa Aesar, 98%, 99-14-9; adipic acid, Sigma, 99%, 124-04-9; acetic acid, Sigma, ACS grade, 64-19-7; pimelic acid, Alfa Aesar; >98%, 111-16-0; succinic acid, TCI, >99%, 110-15-6; tartronic acid, Sigma, >97%, 80-69-3; sodium mesoxalate monohydrate, Chemodex, >98%, 31635-99-1; ᴅʟ-2-hydroxybutyric acid sodium salt, Alfa Aesar, >97%, 5094-24-6; ᴅʟ-3-hydroxybutyric acid sodium salt, Chem Impex Int’l Inc., 100.3%, 150-83-4/306-31-0; ascorbic acid, TCI, >99%, 50-81-7; ammonium nitrate, Fisher, ACS grade, 6484-52-2; sodium nitrate, VWR, ACS grade, 7631-99-4; sodium hydroxide, VWR, ACS grade, 1310-73-2; nitric acid, Sigma, ACS grade, 7697-37-2; and sodium azide, Sigma, 99.8%, 26628-22-8. Lacey carbon, 300 mesh, copper grids (product #01895) from Ted Pella, Inc. were used for electron microscopy.

### Methods

Hexagonal nanoceria particles (4.2 ± 1.2 nm) (mean ± SD) were synthesized using a hydrothermal method [55] and extensively characterized [56], resulting in approximately 2.8 citrate molecules per nm^2^. The product was dialyzed for 120 h at ten times the volume, changing the dialysate every 24 h, against 0.11 M iso-osmotic citric acid adjusted to pH 7.4. The nanoceria sol was then stored in the dark at 4 °C.

Prior to transmission electron microscopy (TEM) imaging, samples were sonicated for 10 min in a sonication bath. Lacey carbon, 300 mesh, copper grids were dipped into the solution for approximately 5 s and dried overnight at room temperature. TEM was used to obtain images of particles throughout the experiment’s duration. The Thermo Scientific Talos F200X instrument equipped with a SuperX G2 EDS detector was operated at an accelerating voltage of 200 keV. The TEM images were recorded on a Ceta CCD camera. DLS was performed using a Brookhaven 90Plus particle size analyzer. Three analysis runs of 5 min each were completed for each sample, and the average result of each run was analyzed and recorded. All samples were evaluated using the multimodal setting.

Nanoceria, 1 mg, was dispersed in 2 mL (500 µg/mL) of 0.11 M iso-osmotic aqueous media at pH 4.5 representative of acidic soil and lysosomes [57–58]. Throughout the experiment the samples were stored in plastic DLS cuvettes, prepared from material that does not significantly absorb UVA radiation [59]. UVA 355 nm radiation at the site of samples exposed to light, at 10 a.m. May 31, 2023, was 6 µW/cm^2^. Sodium hydroxide and nitric acid were used to adjust solution pH and sodium nitrate to adjust the osmolarity. In addition to the 16 carboxylic acids, ascorbic acid, studied by Muhammad et al. [35], was included. A 20 mM sodium salt was used by Dahle and co-workers [32]. This concentration was replicated in this experiment, corresponding to 20 mM NaNO_3_. Ammonium nitrate (20 mM) was also studied since it was used in the initial nanoceria synthesis. Further, DI water was used as a control at pH 6.5. As a bacteriostat, 0.02% sodium azide was added to each sample. Each of the 20 chemicals was studied under two conditions; one sample was placed next to the window for exposure to ambient laboratory light, including natural UV irradiation from the sun, and the other sample was kept in the dark, achieved by aluminum foil covering. Each condition was studied in at least two replicates.

DLS was employed to repeatedly determine the hydrodynamic particle size over 30 weeks (approximately 5040 h). The study of some samples was stopped early because of full dissolution. TEM and particle size/shape analyses were also carried out on citric acid and β-hydroxybutyric acid samples under light and dark conditions after 0, 1, 2, 4, and 8 weeks. High-angle annular dark-field scanning TEM and EDS were used to determine the elemental composition of some particles seen in TEM. In addition, camera photos were taken after 0, 1, 2, and 4 weeks of the samples exposed to citric acid and water under light and dark conditions.

To identify the key functional groups that influence nanoceria agglomeration, the number of carboxylic acid groups and total number of carbons, aliphatic carbons, and hydroxy groups in each carboxylic acid were counted. The molecular ratios of the number of carboxylic acid groups to the total number of carbons and aliphatic carbons, and of the number of hydroxy groups to the total number of carbons and aliphatic carbons were calculated. These values and the position of the carboxylic acid groups in relation to the hydroxy groups were reviewed to suggest the molecular components of carboxylic acids required for stable complexation with nanoceria.

## Supporting Information

Molecular structures of the carboxylic acids, electron microscopy images of nanoceria particles, quantitative DLS results supporting Table 1, and functional group analysis results.

File 1 Additional experimental data.
