# Supplementary material for: Carboxylic acids and light interact to affect nanoceria stability and dissolution in acidic aqueous environments
Source: Beilstein J Nanotechnol. 2023 Jun 27;14:762–80. doi: 10.3762/bjnano.14.63 (PMC10315891; doi:10.3762/bjnano.14.63)
Supplement: File 1 — Additional experimental data. [file Beilstein_J_Nanotechnol-14-762-s001.pdf]

BEILSTEIN JOURNAL OF NANOTECHNOLOGY

Supporting Information

for

Carboxylic acids and light interact to affect nanoceria stability and dissolution in acidic aqueous environments

Matthew L. Hancock, Eric A. Grulke and Robert A. Yokel

Beilstein J. Nanotechnol. **2023,** *14,* 762–780. doi:10.3762/bjnano.14.63

Additional experimental data

License and Terms: This is a supporting information file under the terms of the Creative Commons Attribution License (https://creativecommons.org/licenses/by/4.0). Please note that the reuse, redistribution and reproduction in particular requires that the author(s) and source are credited and that individual graphics may be subject to special legal provisions.
The license is subject to the *Beilstein Journal of Nanotechnology* terms and conditions: (https://www.beilstein-journals.org/bjnano/terms)

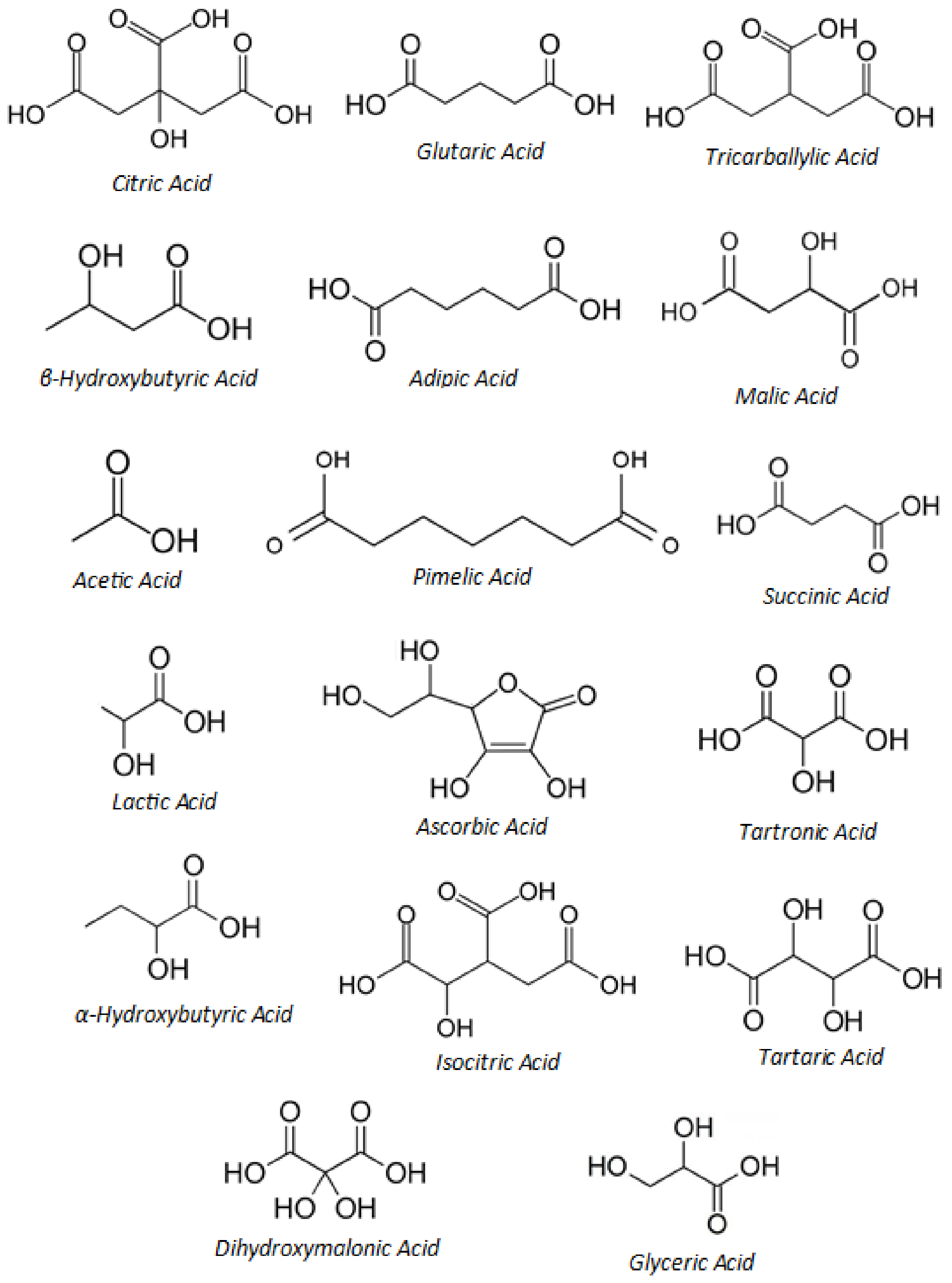

Figure S1: Molecular structures of the sixteen carboxylic acids and ascorbic acid.

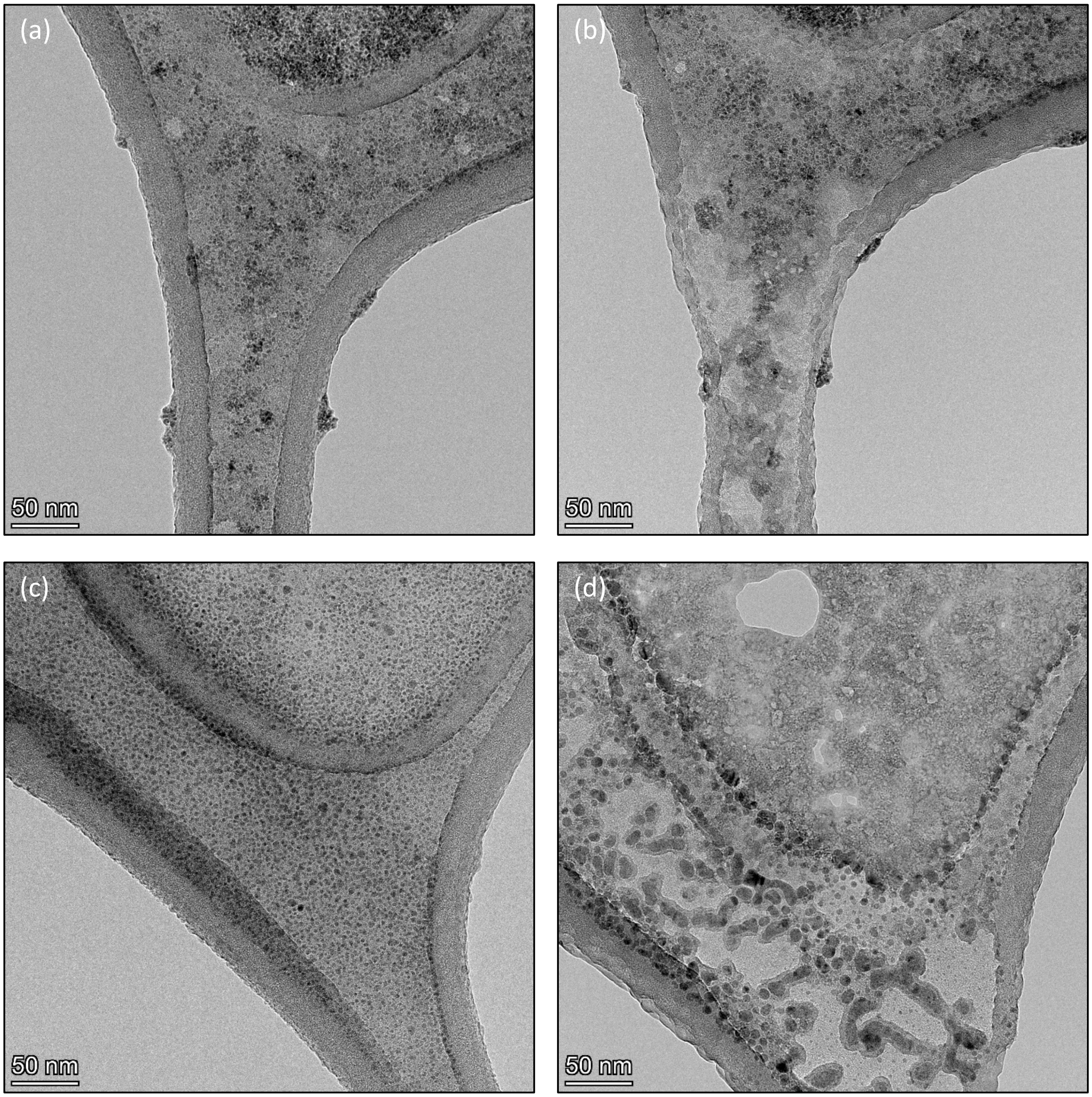

Figure S2: TEM images before and after electron beam exposure upon high magnification. Nanoceria particles exposed to (a) citric acid for two weeks in the dark pre-high magnification; (b) post-high magnification. Nanoceria particles exposed to (c) citric acid for eight weeks in the light pre-high magnification; (d) post-high magnification. The features were altered by the high-intensity electron beam.

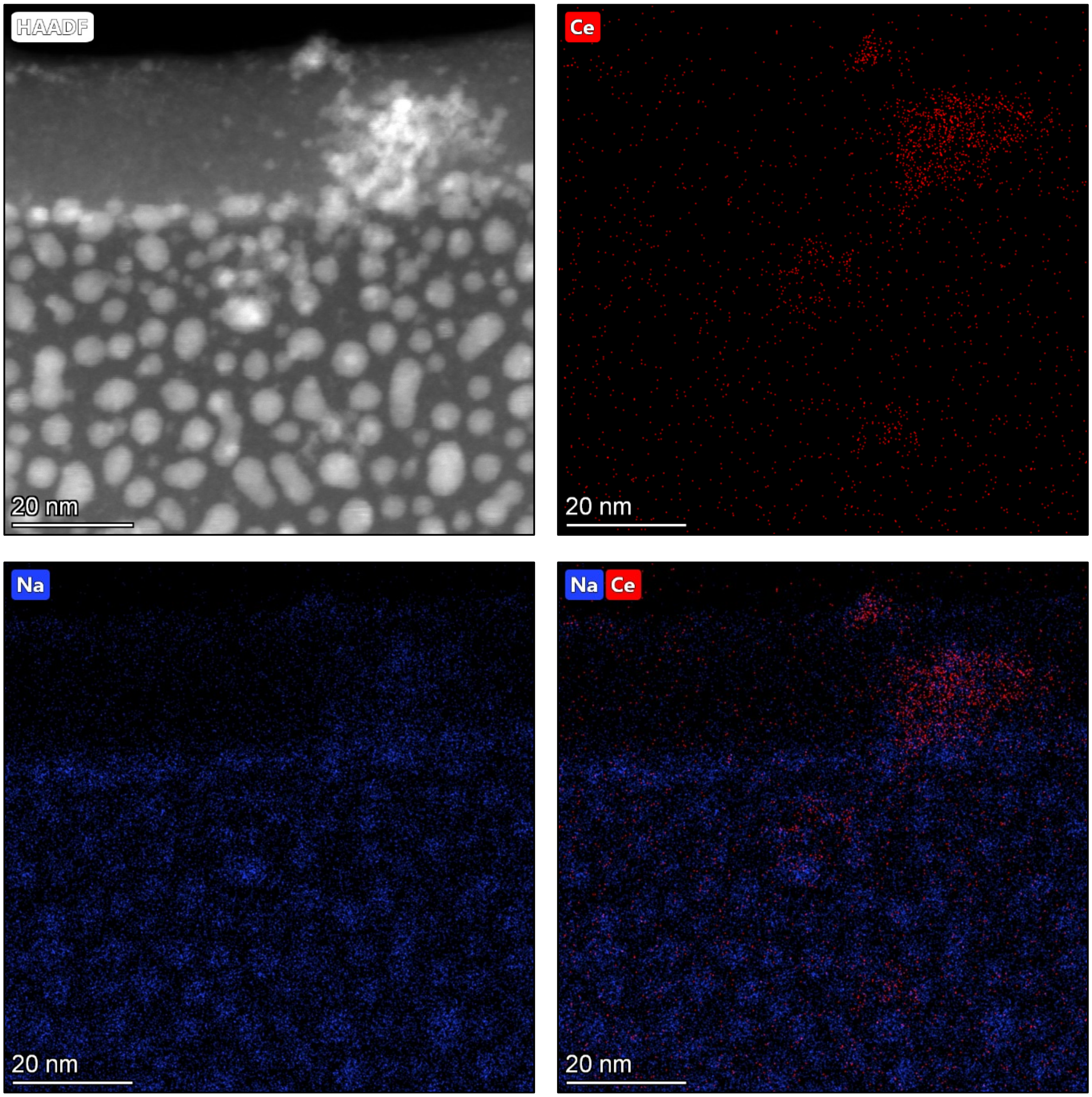

Figure S3: High-angle annular dark-field scanning transmission electron microscopy (HAADF-STEM) and EDS mapping of nanoceria exposed to β-hydroxybutyric acid and light for two weeks. The features contain sodium, but not cerium. The nanoceria appears to localize on the edges of the lacey carbon film as 15–25 nm agglomerates (upper right of the image).

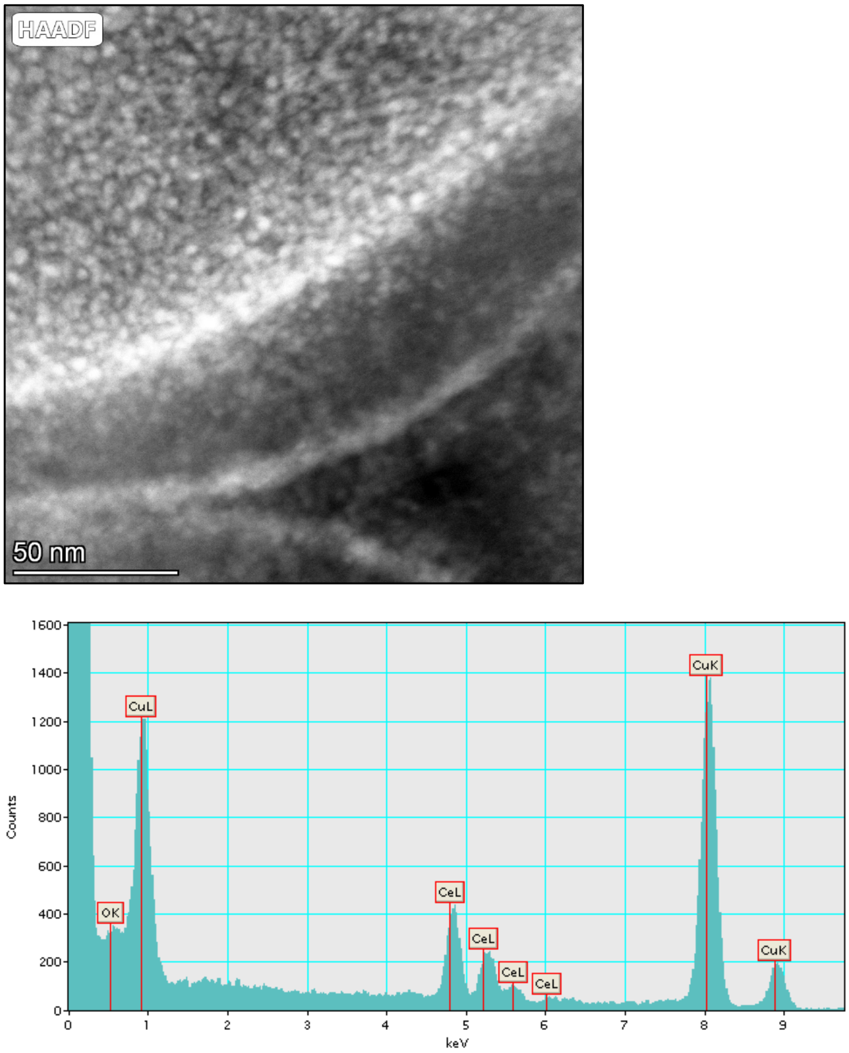

Figure S4: HAADF-STEM image and accompanying EDS map of nanoceria exposed to β-hydroxybutyric acid and light for four weeks. The sample contains cerium and oxygen confirming nanoceria particle agglomeration. Copper is from the sample grid.

Table S1: Quantitative DLS results for nanoceria in the presence of the twenty chemicals under two conditions, grouped by nanoceria stabilization/dissolution category shown in Figure 2 (categories separated by bold lines). Hydrodynamic diameters for each peak were recorded at times shown in the light (left column) and dark (right column) conditions. Two-way ANOVA of the first peak in light for all IDs revealed column factor results of $F(19, 76) = 2.823$, $P = 0.0007$. Tukey multiple comparisons test of column means comparing citric, malic, isocitric, or glyceric acid to all other IDs showed each was significantly different ($P < 0.01$) from β-hydroxybutyric and acetic acids.

		Peak Diameter (nm)			
		Light		Dark	
ID	Time (h)	1st Peak	2nd Peak	1st Peak	2nd Peak
Citric Acid	250	8.4	30.7	7.4	18.7
	500	3.9		7.8	18.0
	1000			7.3	17.3
	3000			5.9	15.9
	5000			6.5	16.6
Malic Acid	250	7.3	16.3	14.0	37.8
	500	1.7	12.3	15.1	35.4
	1000			14.7	29.4
	3000			12.2	23.7
	5000			11.1	25.9
Isocitric Acid	250	10.9	22.4	9.4	21.1
	500	5.6	20.0	11.3	21.7
	1000			11.7	22.1
	3000			14.4	36.8
	5000			13.3	34.4
Glyceric Acid	250	8.4	20.4	11.2	24.5
	500	7.9	19.4	12.1	23.2
	1000	4.9	19.7	12.3	22.6
	3000	10.8	24.4	10.2	21.2
	5000	14.5	32.7	10.5	22.0
Lactic Acid	250	9.3	20.9	10.3	21.3
	500	9.5	20.4	9.0	21.4
	1000	10.7	20.5	6.9	20.4
	3000	2014	9399	4.6	19.0
	5000	913	2412	3.9	19.0
Tartaric Acid	250	6.7	18.6	12.2	24.1
	500	4.4	19.1	14.2	26.6
	1000	3.4	19.9	10.8	21.7
	3000	656	9040	15.6	
	5000	883	9140	16.0	
NH_4NO_3	250	5.7	19.4	12.6	25.0
	500	6.3	19.7	14.0	27.6
	1000	21.1	48.2	11.6	20.9
	3000	2486	9479	6.5	18.9
	5000	1262	9262	7.9	17.8
Water	250	5.9	19.8	14.3	34.0
	500	5.0	19.7	14.3	33.4
	1000	10.6	21.3	14.5	30.2
	3000	493	7285	15.6	34.6
	5000	275	6606	15.1	31.0
α-Hydroxy-butyric Acid	250	9.7	20.6	12.7	24.9
	500	14.9	76.0	13.2	24.8
	1000	751	2047	11.4	21.7
	3000	1776	6711	9.9	21.7
	5000	1291	4475	10.9	20.9
β-Hydroxy-butyric Acid	250	11.7	23.4	11.1	22.9
	500	29.1	92.5	8.8	21.3
	1000	2353	8947	8.8	21.1
	3000	2189	9432	11.7	23.0
	5000	1705	8154	5.4	19.5

		Peak Diameter (nm)			
		Light		Dark	
ID	Time (h)	1st Peak	2nd Peak	1st Peak	2nd Peak
Succinic Acid	250	12.2	23.5	15.1	28.5
	500	180.8	956	9.1	21.3
	1000	1553	5961	8.1	19.7
	3000	1028	4337	8.0	18.8
	5000	614	2591	4.7	18.0
Pimelic Acid	250	20.8	46.7	13.1	26.2
	500	658	2416	11.8	22.7
	1000	275	2266	16.2	33.8
	3000	1181	4817	13.4	29.8
	5000	1013	4226	79.2	2673
Glutaric Acid	250	16.8	35.8	6.6	17.4
	500	1369	4767	6.6	18.4
	1000	806	3560	6.4	17.4
	3000	961	4104	25.6	106.7
	5000	643	3097	809	3072
Tricarbal-lylic Acid	250	107	536	6.4	19.1
	500	1325	4794	6.9	19.0
	1000	1673	4983	6.0	18.1
	3000	388	1536	10.2	20.9
	5000	692	2764	302	1461
Adipic Acid	250	12.3	22.6	10.3	19.6
	500	1105	4663	9.1	19.4
	1000	774	2195	9.3	18.3
	3000	793	2877	19.4	49.1
	5000	910	3841	831	3047
Acetic Acid	250	10.8	20.9	15.7	28.8
	500	1224	3817	7.8	18.1
	1000	1365	4222	11.3	20.3
	3000	1666	9358	14.5	44.0
	5000	1696	9340	748	2121
$NaNO_3$	250	13.2	38.0	13.8	26.7
	500	199	3919	9.1	18.4
	1000	662	2763	7.9	18.2
	3000	416	3111	8.1	18.4
	5000	409	6330	8.7	17.8
Tartronic Acid	250	633	1704	1070	6248
	500	1429	8056	1134	7779
	1000	1114	7836	680	9052
	3000	661	9075	526	6566
	5000	674	2207	209	1508
Dihydoxy-malonic Acid	250	485	2792	357	1770
	500	1822	6850	845	4078
	1000	1081	7102	1811	8768
	3000	640	2939	861	5679
	5000	222	2006	1200	4631
Ascorbic Acid	250	113	486	60.4	161
	500	1385	7961	1142	7066
	1000	1215	7765	1277	5488
	3000	1646	8349	1525	5987
	5000	85.6	613	168	401

Table S2: The number of carboxylic acid groups (CA), total number of carbons (C), aliphatic carbons (AC), and hydroxy groups (OH) in each molecule are shown. The molecular ratios were calculated and used to compare amongst each other. The acids that totally dissolved nanoceria in the presence of light (citric, malic, and isocitric) had at least two CA and three OH groups, and a ratio greater than or equal to 1½ CA:AC and 2 OH:AC. These numbers and ratios were greater than the other acids in this study.

					Molecular Ratios			
ID	CA	C	AC	OH	CA:C	CA:AC	OH:C	OH:AC
Citric Acid	3	6	2	4	1/2	1 1/2	2/3	2
Malic Acid	2	4	1	3	1/2	2	3/4	3
Isocitric Acid	3	6	2	4	1/2	1 1/2	2/3	2
Glyceric Acid	1	3	0	3	1/3		1	
Lactic Acid	1	3	1	2	1/3	1	2/3	2
Tartaric Acid	2	4	0	4	1/2		1	
α-Hydroxybutyric Acid	1	4	2	2	1/4	1/2	1/2	1
β-Hydroxybutyric Acid	1	4	2	2	1/4	1/2	1/2	1
Succinic Acid	2	4	2	2	1/2	1	1/2	1
Pimelic Acid	2	7	5	2	2/7	2/5	2/7	2/5
Glutaric Acid	2	5	3	2	2/5	2/3	2/5	2/3
Tricarballylic Acid	3	6	3	3	1/2	1	1/2	1
Adipic Acid	3	6	4	2	1/2	3/4	1/3	1/2
Acetic Acid	1	2	1	1	1/2	1	1/2	1
Tartronic Acid	2	3	0	3	2/3		1	
Dihydroxymalonic Acid	2	3	0	4	2/3		1 1/3	
